# Analysis of wild-species introgressions in tomato inbreds uncovers ancestral origins

**DOI:** 10.1186/s12870-014-0287-2

**Published:** 2014-10-28

**Authors:** Naama Menda, Susan R Strickler, Jeremy D Edwards, Aureliano Bombarely, Diane M Dunham, Gregory B Martin, Luis Mejia, Samuel F Hutton, Michael J Havey, Douglas P Maxwell, Lukas A Mueller

**Affiliations:** Boyce Thompson Institute for Plant Research, 533 Tower Rd, Ithaca, NY 14853 USA; Department of Plant Pathology and Plant-Microbe Biology, Cornell University, Ithaca, NY 14853 USA; Facultad de Agronomía, Universidad de San Carlos de Guatemala, Guatemala City, 01012 Guatemala; University of Florida, Gulf Coast Research and Education Center, 14625 CR 672, Wimauma, FL 33598 USA; USDA-ARS Department of Horticulture, University of Wisconsin, 1575 Linden Drive, Madison, WI 53706 USA; Department of Plant Pathology, University of Wisconsin-Madison, Madison, WI 53706 USA

**Keywords:** *Solanum lycopersicum*, *Solanum pimpinellifolium*, *Solanum chilense*, Genomic introgressions, Genome sequencing, Disease resistance, Single nucleotide polymorphism, Wild species, Domestication, Phylogenetics

## Abstract

**Background:**

Decades of intensive tomato breeding using wild-species germplasm have resulted in the genomes of domesticated germplasm (*Solanum lycopersicum*) being intertwined with introgressions from their wild relatives. Comparative analysis of genomes among cultivated tomatoes and wild species that have contributed genetic variation can help identify desirable genes, such as those conferring disease resistance. The ability to identify introgression position, borders, and contents can reveal ancestral origins and facilitate harnessing of wild variation in crop breeding.

**Results:**

Here we present the whole-genome sequences of two tomato inbreds, Gh13 and BTI-87, both carrying the begomovirus resistance locus *Ty-3* introgressed from wild tomato species. Introgressions of different sizes on chromosome 6 of Gh13 and BTI-87, both corresponding to the *Ty-3* region, were identified as from a source close to the wild species *S. chilense*. Other introgressions were identified throughout the genomes of the inbreds and showed major differences in the breeding pedigrees of the two lines. Interestingly, additional large introgressions from the close tomato relative *S. pimpinellifolium* were identified in both lines. Some of the polymorphic regions were attributed to introgressions in the reference Heinz 1706 genome, indicating wild genome sequences in the reference tomato genome.

**Conclusions:**

The methods developed in this work can be used to delineate genome introgressions, and subsequently contribute to development of molecular markers to aid phenotypic selection, fine mapping and discovery of candidate genes for important phenotypes, and for identification of novel variation for tomato improvement. These universal methods can easily be applied to other crop plants.

**Electronic supplementary material:**

The online version of this article (doi:10.1186/s12870-014-0287-2) contains supplementary material, which is available to authorized users.

## Background

A priority in modern plant breeding is the introduction of novel variation for desirable traits; Biotic and abiotic stresses are the most crucial to increase yield and provide reliable food production. Tomato (*Solanum lycopersicum*) is an important food crop and a model species for studying processes such as fleshy fruit ripening, fruit development [1], and the molecular basis of disease resistance [2,3].

Tomato originated in the South American Andean mountains, deserts, and coastal plains [4]. During the domestication of tomato from its ancestral wild species, the tomato genome went through a genetic bottleneck, reducing its genetic diversity to less than 5% of the diversity found in its closest wild relatives [5,6]. Moreover, human selection for traits related to yield and fruit qualities, such as size, weight, color, sugar content, and shelf life, has disregarded disease resistance traits. Consequently, tomato heirloom cultivars are susceptible to many pathogens, including bacteria, viruses, fungi, nematodes and insect pests, and resistance alleles are present only in wild tomato relatives [7]. Since these species can be outcrossed with cultivated ones, breeders have introgressed wild genomes into cultivated varieties since 1917 [8,9], a practice that continues today [7]. Most disease resistance genes have been introgressed from wild species such as *Solanum chilense* [10-12], *S. peruvianum* [13-15], S*. habrochaites* [16], *S. pennellii* [17], and *S. pimpinellifolium* [7,18].

Begomoviruses cause major diseases affecting tomatoes in tropical and subtropical regions. Symptoms vary, but all involve some level of leaf distortion and reduction of growth and yield [19-21]. Management strategies for control of begomovirus-incited tomato diseases have traditionally focused on the insect vector [22]. For begomovirus resistance, at least four loci have been introgressed into tomato from three accessions of *S. chilense* and *S. habrochaites* [11,16,21,23].

The release of the reference tomato genome sequence (variety Heinz 1706) in early 2012 has enabled a multitude of new genetic and genomic approaches [24], such as mapping reads from re-sequenced breeding lines. Using the mapping approach, genome regions that contain a limited number of SNPs can be efficiently aligned to the reference sequence, and using paired-end sequencing, insertions and deletions can be detected. However, large insertions and regions that are highly divergent cannot easily be characterized using this mapping approach. More high quality de novo assemblies of reference genomes, especially of wild germplasm, are required for the analysis of re-sequenced genome regions that cannot be mapped using the existing resources [25].

Since virtually all tomato disease resistance genes originate from wild relatives, further knowledge of these genomes will facilitate introgression of multiple disease resistances into elite cultivars. Also, while all tomato species share largely syntenic genomes and can outcross, the genome content of the reference genome is not completely identical even to other commercial tomato cultivars. For example, the fruit shape gene *SUN* has been duplicated in some varieties, but its functional copy is not present in Heinz 1706 (H1706) [26]. Another example is the bacterial resistance gene *Pto*, which was introgressed from the wild tomato species, *S. pimpinellifolium*, in the 1930’s and later positionally cloned [2,27]. A functional version of this gene is also missing in H1706.

Introgression of wild-species genomic regions into domesticated species is a widely used practice for increasing diversity in tomato as well as other crop species [28]. After several generations of backcrossing and selection, larger introgressions carrying favorable traits, as well as cryptic introgressions, are present throughout the genome. While excellent genetic maps exist for tomato [29], many of the available maps are not very dense and do not allow the precise definition of introgression points. The selection process can be accompanied by linkage-drag, producing genomes with tightly linked detrimental alleles, which require many rounds of backcrossing and fine-mapping to eliminate [30]. Thus, the ability to define the borders and contents of wild-species introgressions can contribute significantly to reducing the number of generations required for selecting favorable alleles while minimizing negative variation. Identification of introgressions can help to identify candidate genes responsible for beneficial traits such as disease resistance [31].

Other crops, such as maize, rice, barley [32], bean [33], and melon [34], exhibit wild introgression patterns similar to those found in tomato. These genomes, and those of tomatoes [35], have been studied recently using high-density SNP chips. However, while these technologies are excellent in detecting traits in populations and revealing population structure [36], they are less informative in defining introgression borders and their content. On the other hand, the whole-genome sequencing approach provides more detailed information on genic content and the origins of the introgressed regions through comparison to genomes of wild species involved in the breeding process [37]. Other work related to re-sequencing tomato genomes was published recently, and demonstrates how SNP calling in lines of domesticated tomatoes can reveal substantial differences between domesticated accessions due to wild introgressions [38]. Re-sequencing of tomato accessions has also been used in genome-wide association studies (GWAS) for associating SNPs with agronomically important traits [39].

For this study, two begomovirus-resistant inbreds were chosen, Gh13 [40] and BTI-87 (D.P. Maxwell, unpublished data), which are presumed to originate from different accessions. Gh13 was developed in Guatemala [41] were it has been tested over multiple seasons and consistently shows very good resistance to high begomovirus pressure. Resistance in Gh13 was, until now, presumably derived from *S. habrochaites* [42]. BTI-87 was also developed in Guatemala and maintains a high level of resistance derived from the begomovirus-resistant inbred Gc171, which is in turn derived from *S. chilense* accession LA1932 [43]. Both inbred lines carry a *Ty-3* resistance allele, as well as several other resistance genes from several wild accession sources.

**Figure 1:**
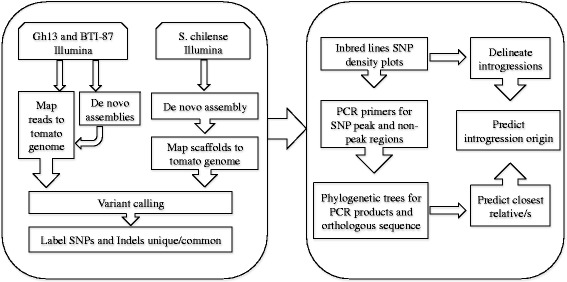
**Schematic view of the genome assembly and the introgression detection pipelines.**

We used whole-genome sequencing (WGS) to detect introgressions from wild species in two begomovirus-resistant inbreds. The boundaries of the introgressions were established and the source of several introgressions was determined (Figure 1). The findings provide insight into the genome structure of tomato inbreds derived from a breeding program, and demonstrate how breeding can greatly benefit from WGS, which can diminish time consuming phenotypic screening.

## Results

### Sequencing and assembly

Paired-end libraries of the Gh13 and BTI-87 genomes were each sequenced in one Illumina HiSeq lane. Mapping the Gh13 genome to the reference tomato H1706 genome yielded 14.7× coverage of the H1706 genome, after removing low quality reads and duplicates, with 97.6% coverage of the reference genome. Gaps in the Gh13 genome were estimated to span 9.2 Mb, and the total number of SNPs was 288,640 (Table 1). The BTI-87 genome mapping to the reference tomato genome yielded coverage of 32.3×, represented 96.5% of the H1706 genome, with 79.9 Mb of gaps in the assembly, and 702,560 SNPs (Table 1), and 77,652 shared SNPs with Gh13, compared to the reference tomato genome.

**Table 1:** **Reference-guided assembly metrics**

	**Heinz 1706^**	**LA1589**	**Gh13**	**BTI-87**	LA1932*
Filtered reads in millions	462.7	281.5	392.9	402.267066	
Mapped reads (% mapped)	426.1 (92.1%)	247.7 (88%)	385.4 (98%)	380.9 (94.7%)	
Coverage depth	39.3	25	14.7	32.3	
Coverage of tomato genome	0.992	0.95	0.976	0.965	
Number of gaps (Mb)	76,276 (5.9)	209,919 (38.9)	90,727 (9.2)	165,894 (79.9)	
Gaps >500 bp	1,660	14,396	3,058	19,479	
Gaps >5000 bp			247	286	
SNPs		2,753,307 (0.35%)	288,640 (0.037%)	702,560 (0.09%)	8,123,431 (1%)
Indels		437,943	69,289	130,029	718,185

^Subset of the available libraries for comparison purposes.

*Low coverage reference-based assembly.

LA1589 (*S. pimpinellifolium*) and LA1932 (*S. chilense*).

The major difference in coverage depth between lines Gh13 and BTI-87 (14.7× and 32.3×, respectively) was attributed to the quality of the genomic DNA. The DNA library of BTI-87 was of higher quality than the one of Gh13, in that it contained fewer exact-duplicate reads. The difference in coverage did not affect the ability to map the reads to the reference genome and to call SNPs with high confidence using the same criteria. These genomes yielded similar genome coverage levels (97.6% and 96.5%), but the coverage in Gh13 is slightly higher since it has fewer SNPs and gaps than BTI-87, mainly due to fewer regions of introgressions from wild species.

Both Gh13 and BTI-87 genome sequences are available on the Sol Genomics Network (SGN; http://solgenomics.net). Positions of SNPs in both genomes can be found in the Genome Browser track, and can be used for designing new markers.

### SNP distribution

**Figure 2:**
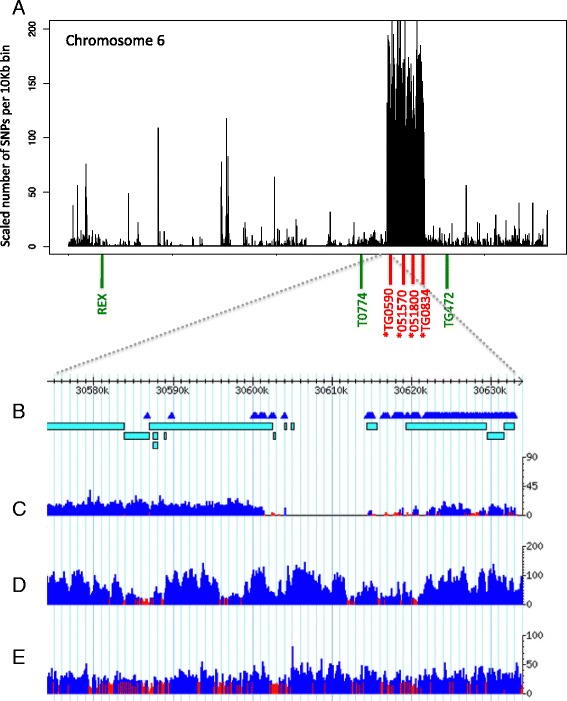
**SNP density and coverage plots for chromosome 6. A)** SNP density plot of the Gh13 chromosome 6. Peak region on chromosome 6 around 30.6 Mb–34.24 Mb. (*) Denotes PCR markers within the SNP peak region. **B)** Visualization of the 50-Kb region around the beginning of the SNP peak region (30.58–30.63 Mb). SNP marks are denoted in triangles. Bars represent de novo scaffolds of Gh13. **C)** Illumina coverage plot of the Gh13 genome mapped to the reference H1706 genome **D)** coverage of the H1706 genome **E)** coverage of the S. pimpinellifolium genome. Y axes for plots C-E represent number of Illumina reads mapped in that region.

The large SNP density peak region on chromosome 6 in Gh13, which spans the position of the *Ty-3* region [21] (30.6–34.22 Mb; Figure 2A; Additional file 1: Figure S1), shows that this SNP analysis methodology can effectively identify introgressed genomic regions. Moreover, we identified an introgression in line BTI-87 that has the *Ty-3a* locus from *S. chilense* LA1932. BTI-87 has a similar SNP density peak on chromosome 6, spanning a smaller region of 1.33 Mb around the *Ty-3* locus region (30.81–32.14 Mb. Additional file 2: Figure S2).

**Figure 3:**
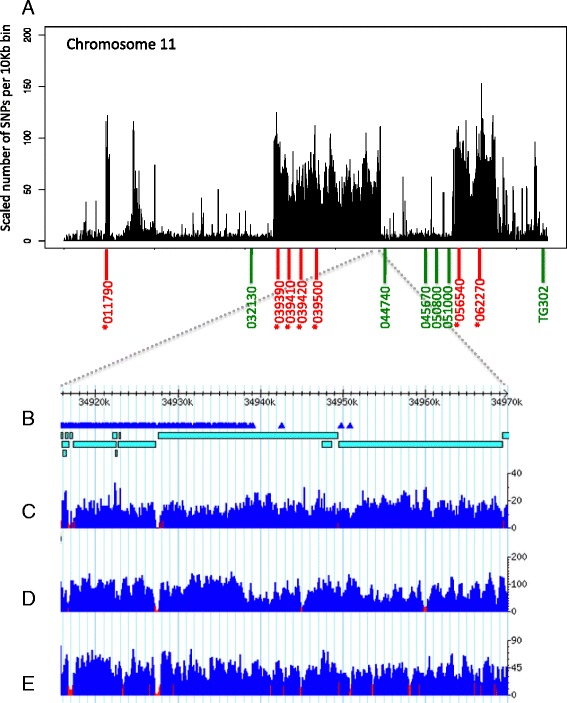
**SNP density and coverage plots for chromosome 11. A)** SNP density plot of the Gh13 chromosome 11. (*) Denotes PCR markers within the three assayed SNP peak regions (4.58–5.01 Mb, 23.12–34.94 Mb, 42.89–47.79 Mb). **B)** Visualization of the 50-Kb region around the end of the largest SNP peak region (34.92–34.97 Mb). SNP marks are denoted in triangles. Bars represent de novo scaffolds of Gh13. **C)** Illumina coverage plot of the Gh13 genome mapped to the reference H1706 genome **D)** coverage of the H1706 genome **E)** coverage of the S. pimpinellifolium genome. Y axes for plots C-E represent the number of Illumina reads mapped in that region.

We also identified a number of other distinct regions of SNP density peaks across the entire Gh13 genome, the most notable of which is apparent on chromosome 11, with two large peak regions spanning 11.76 Mb (23.18–34.94 Mb) and 4.49 Mb (43.18–47.67 Mb) (Figure 3A; Additional file 3: Table S1). Other notable SNP peak regions were identified on chromosome 4 (2.17 Mb and 2.11 Mb), chromosome 7 (1.29 Mb), and chromosome 10 (1.79 Mb). Other candidate SNP peak regions were identified on all chromosomes, ranging in length between 50 Kb to 11.76 Mb (Table 2). We defined a SNP peak as a region having 10 SNPs or more in five or more continuous 10-Kb windows, allowing gaps of up to 40 Kb, to include regions that may have low coverage due to insufficient number of reads or inability to map to the region in the reference genome, while not allowing maximum gap size to exceed the minimum SNP-peak size of 50 Kb. Our goal was to test whether it is possible to reveal relatively small introgressions by defining a minimum window size as small as 50 Kb. Using the criteria of 150 Kb used in the H1706 genome analysis [24], would yield only 32 SNP-peak regions in Gh13 and overlooking many regions of significantly high number of SNPs. To test the cutoff for selecting minimum number of SNPs per 10 Kb window for defining SNP-peak regions we calculated the average number of SNPs per 10 Kb window in the entire genome of Gh13 and compared it to the average number of SNPs in the non-peak regions when calling peak regions using a minimum number of 3, 5, 10, 15, and 20 SNPs per 10 Kb. Our statistical analysis shows the average number SNPs in the entire genome is not significantly different from the non-peak regions when using minimum number of 3 and 5 SNPs (p < 0001, p = 0.0026), but is significantly higher when using 10, 15, and 20 SNPs per 10 Kb window (p = 0.2152, p = 0.4009, p = 0.8383). Therefore we chose a minimum value of 10 SNPs per 10 Kb window, which provides statistical confidence for distinguishing SNP-peak regions from non-peak regions. For testing the reference value of minimum number of SNPs per 10 Kb window in line BTI-87 we have excluded chromosomes 4 and 9, since these have very large SNP peaks covering more than 70% in each of the two chromosomes. The statistical analysis of the remaining 10 chromosomes of BTI-87 shows similar results to the statistical analysis of the Gh13 genome (minimum of 3 and 5 SNPs; p = 0.0003, p = 0.0106. Minimum of 10, 15, and 20 SNPs; p = 0.1793, p = 0.6284, p = 0.6909).

**Table 2:** **Introgression metrics for Gh13 and BTI-87**

	**Gh13**	**BTI-87**
Number of introgressions	144	146
Introgressions in Heinz 1706	60	37
Total size (Mb)	49.42	150.16
SNPs in introgressions	171,711	641,454
Gene models in introgressions	2,326	5,633
Smallest introgression (Kb)	50	50
Largest introgression (Kb)	11,760	42,870
Average introgression size (Kb)	343	1,028
Median introgression size (Kb)	130	200

The total number of SNP-peak regions identified using these criteria was 144, spanning 49.42 Mb with a total of 171,711 SNPs, of which 94 regions were 100 Kb or larger (Table 2; Additional file 3: Table S1). Using the same criteria for calling SNP peaks in BTI-87, we also detected 146 regions in its genome, spanning 150.16 Mb with a total of 641,454 SNPs (Table 2; Additional file 4: Table S2). The SNP peak flanking the *Ty-3* locus region on chromosome 6 is 1.33 Mb. A striking difference between SNP-distribution in the two genomes is the large introgressions detected in chromosomes 4, 6, and 9 of BTI-87 (total of 48.89 Mb in 11 regions in chromosome 4, 18.51 Mb in 47 regions in chromosome 6, and 53.39 Mb in 10 regions in chromosome 9).

### Detection of putative introgressions

To identify potential introgressions, we identified SNPs between Gh13 and the reference genome, and discovered regions that were significantly different from the reference genome (tomato SL2.40 genome build, http://solgenomics.net/organism/Solanum_lycopersicum/genome). These regions could indicate introgressions in either the analyzed genome or in the reference genome. By plotting the number of SNPs in the Gh13 and BTI-87 genomes in windows of 10 Kb, a number of regions across the genome that could be potential introgressions from wild species were identified (Additional file 1: Figure S1, Additional file 2: Figure S2).

To test the hypothesis that regions with high SNP density correspond to introgressions from wild species, the SNPs between each of the inbred lines, Gh13 and BTI-87, and the reference tomato genome were compared to SNPs in the genomes of *S. pimpinellifolium* LA1589 [24], and the heirloom line Yellow Pear (YP). *S. pimpinellifolium* is a close relative of the domesticated tomato species, *S. lycopersicum* [4], and the reference tomato genome, H1706, has a *S. pimpinellifolium* parent in its background [24,44]. Therefore, we expected to find regions of introgressions from *S. pimpinellifolium* in the reference tomato genome, and perhaps from other wild species. YP does not show any traces of introgressions from wild species [37]. Thus any regions displaying a high density of SNPs between YP and H1706 could indicate regions in H1706 that did not originate from *S. lycopersicum*, and were likely introgressed during the breeding of this line [24,44]. The SNP density plots of both Gh13 and BTI-87 display regions with major differences between each genome and the reference tomato genome, but it is impossible to determine from this information alone whether the SNP peak represents an introgression in the inbred line or in the H1706 genome. By determining SNPs shared between Gh13 and *S. pimpinellifolium*, it is possible to predict which introgressions in Gh13 are most likely from *S. pimpinellifolium*. SNP peak regions that are shared between Gh13 and YP (Gh13 X YP) but different in H1706 (H1706 X Gh13 and H1706 X YP) most likely represent wild introgressions in the H1706 genome.

The SNP peak regions in Gh13 that do not correspond to peaks in the YP or to the *S. pimpinellifolium* genome, can be designated as introgressions in Gh13 originating from a different wild species (Additional file 3: Table S1). H1706 is not introgression-free, containing introgressions from *S. pimpinellifolium* [24,44] and possibly other wild accessions. We have detected in Gh13 SNP-peak regions that share SNPs with YP (60 out of the 144 detected candidate introgression regions). Since YP has no wild introgressions and is considered to have 100% *S. lycopersicum* genome [37] we can conclude these regions in the inbred Gh13 correspond to the introgression-free *S. lycopersicum* genome (Additional file 3: Table S1; Table 2). For example, on chromosome 10 of Gh13, 5.18 Mb in 15 SNP peak regions are shared with YP and not shared with *S. pimpinellifolium*, indicating all these regions are introgressions from unknown wild species in H1706 which were not recorded in its pedigree [44]. Pedigree origins are also not always reliable, as we have demonstrated with the *Ty-3* gene in line Gh13, which was reported to have *S. habrochaites* as the source of resistance, but the *Ty-3* locus was introduced from *S. chilense*, which is not recorded in the line’s pedigree.

**Figure 4:**
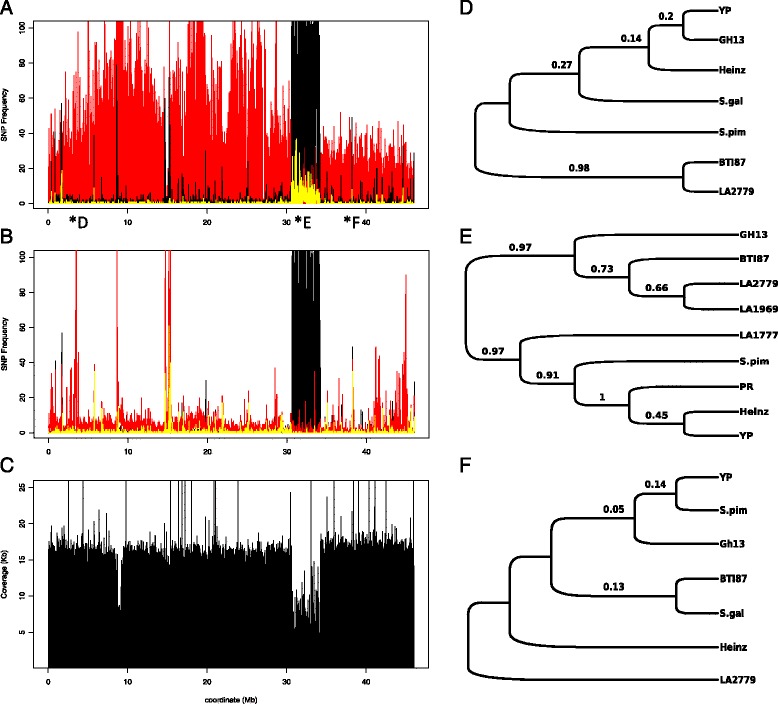
**Chromosome 6 SNPs and gene trees of line Gh13 compared to selected tomato wild species and accessions. A)** Chromosome 6 SNP plots of inbred line Gh13 (black) and S. pimpinellifolium (red) compared to H1706. Shared SNPs are denoted in yellow. **B)** Chromososme 6 SNP plots of inbred line Gh13 (black) and heirloom line YP (red) compared to H1706. Shared SNPs are denoted in yellow. **C)** Coverage plot of chromosome 6 of Gh13. **D)** Gene tree of non-peak region (marker REX). **E)** Gene tree of SNP peak region (marker TG590). **F)** Gene tree of non-peak region (marker TG472).

**Figure 5:**
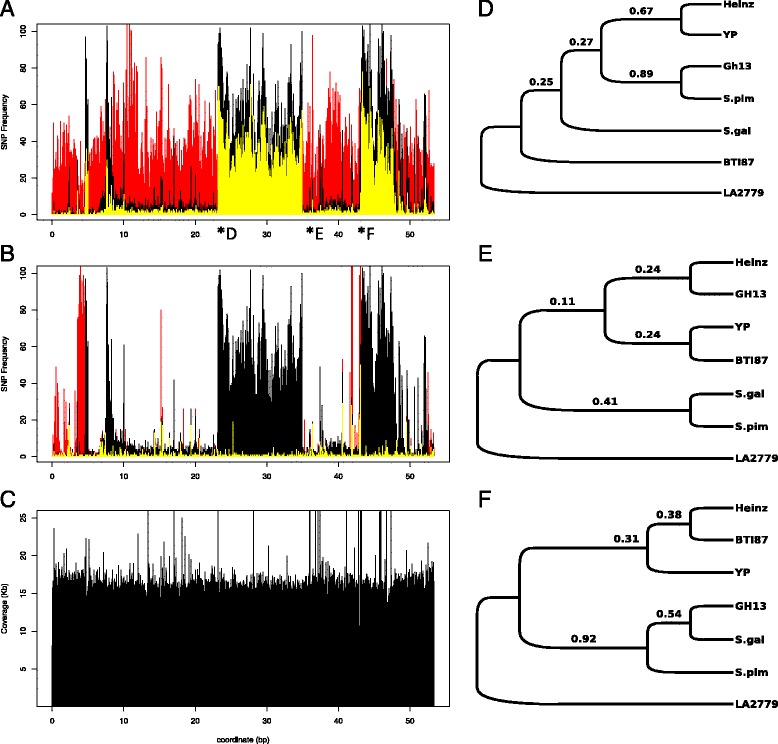
**Chromosome 11 SNPs and gene trees of line Gh13 compared to selected tomato wild species and accessions. A)** Chromosome 11 SNP plots of inbred line Gh13 (black) and S. pimpinellifolium (red) compared to H1706. Shared SNPs are denoted in yellow. **B)** Chromosome 11 SNP plots of inbred line Gh13 (black) and heirloom line YP (red) compared to H1706. Shared SNPs are denoted in yellow. **C)** Coverage plot of chromosome 11 of Gh13. Gene trees of three regions from chromosome 11. **D)** Gene tree of SNP peak region (marker P11-039390). **E)** Gene tree of nonpeak region (marker P11-050800). **F)** Gene tree of SNP-peak region (marker P11-062270).

The SNP peak detected in chromosome 6 of Gh13 (Figure 2A) and BTI-87 (Additional file 2: Figure S2) shows no significant overlap either with SNPs of *S. pimpinellifolium* or with those of YP, indicating these are introgressions of a wild species other than *S. pimpinellifolium* (Figure 4A; Additional file 3: Table S1). Chromosome 11 of line Gh13 shows three distinct regions which we conclude are introgressed from *S. pimpinellifolium*, because the majority of the SNPs are shared between the two (Figure 5A). In contrast, the SNP introgressions in chromosome 11 of BTI-87 are different than those in Gh13 (Additional file 2: Figure S2; Additional file 3: Table S1, Additional file 4: Table S2).

On chromosome 4 of Gh13 we detected a large 2.17-Mb introgression (from 53.35 Mb to 55.52 Mb), which is closest to *S. pimpinellifolium*. However, this introgression includes a few fragments that range in size between 10 and 200 Kb for which YP has a significant number of matching SNPs (more than 10 SNPs in 10 Kb). The second largest SNP peak in chromosome 4 shows similarity to *S. pimpinellifolium* from 57.53 Mb to 57.91 Mb, immediately followed by 1.73-Mb region (57.91 Mb to 59.64 Mb) that most likely corresponds to an introgression in H1706 due to the high SNP density shared between Gh13 and YP (Additional file 3: Table S1). In some of those regions of high SNP density in YP, it is unclear as to the origin of introgression in Gh13 (Additional file 3: Table S1). Further phylogenetic analysis is required for each of those regions to clarify its origins.

### PCR sequencing and gene trees

To investigate the origin of each detected SNP peak region on chromosomes 6 and 11 of Gh13, PCR primers were designed for amplifying fragments outside and inside the selected SNP peak regions (Figures 2A, 3A). PCR sequences were aligned, analyzed for SNPs (Table 3) and indels, and used for building phylogenetic gene trees including sequences from H1706, the heirloom lines YP and Purple Russian (PR), the inbred lines Gh13 and BTI-87, and the wild species *S. pimpinellifolium*, *S. galapagense*, *S. chilense*, and *S. habrochaites*.

**Table 3:** **PCR primers and fragment sequencing results**

**Marker**	**GenBank number**	**Position**	**SNP region^**	Heinz*	YP*	**PR**	**Gh13**	LA1589*	**LA2779**	**LA1969**	**LA1777**	**LA0386**	**BTI-87**	***S. gal*** *
REX	KF887310, KF887311	2,633,235	Chr6 NP	a	a	-	a	a	b	-	-	-	b	a
T0774	KF887301, KF887302	30,027,677	Chr6 NP	a	ac	-	a	ac	d	-	-	-	ad	ab
TG590	KF887295–KF887300	31,166,442	Peak Chr6	a	a	ab	be	ac	b	b	ad	d	bf	-
P6-051570	KF887303–KF887307	31,568,208	Peak Chr6	a	a	a	b	a	b	b	c	c	b	a
T0834	KF887312–KF887316	33,353,915	Peak Chr6	a	a	a	c	ab	cd	c	e	-	a	a
TG472	KF887308, KF887309	37,982,169	Chr6 NP	a	a	-	a	a	c	-	-	-	a	ab
P11-011790	KF887317, KF887318	4,777,374	Peak Chr11	a	a	-	b	b	c	-	-	-	b	a
P11-032130	KF887319, KF887320	21,629,704	Chr11 NP	a	a	-	a	ab	c	-	-	-	a	ab
P11-039390	KF887321, KF887322	23,182,355	Peak Chr11	a	a	-	c	c	d	-	-	-	a	ab
P11-039410	KF887323, KF887324	23,342,156	Peak Chr11	a	a	-	b	b	-	-	d	-	a	bc
P11-039420	KF887325	23,390,919	Peak Chr11	a	a	-	b	b	-	-	-	-	a	bc
P11-039500	KF887326	24,113,034	Peak Chr11	a	a	-	b	b	-	-	-	-	a	c
P11-044740	KF887327, KF887328	36,050,109	Chr11 NP	a	a	-	a	a	b	-	-	-	a	a
P11-045670	KF887329, KF887330	40,368,253	Chr11 NP	a	a	-	a	a	b	-	-	-	a	a
P11-050800	KF887331, KF887332	41,218,579	Chr11 NP	a	a	-	a	b	c	-	-	-	a	b
P11-051000	KF887333, KF887334	42,147,976	Chr11 NP	a	a	-	a	ab	c	-	-	-	a	ac
P11-056540	KF887335	43,330,076	Peak Chr11	a	a	-	b	b	-	-	-	-	a	bc
P11-062270	KF887336, KF887337	46,239,133	Peak Chr11	a	a	-	b	b	c	-	-	-	a	b
TG0302	KF887338–KF887341	51,878,967	Chr11 NP	a	a	-	a	b	c	-	d	d	a	b

^NP - Non SNP-peak.

*Heinz 1706, Yellow Pear, *S. galapagense*, and *S. pimpinellifolium* (LA1589) sequences were extracted from their genome assemblies.

On chromosome 6, the three selected regions outside the SNP peak (markers REX, T0774, TG472; Figure 2A) showed, as expected, that the Gh13 sequence was identical to the sequences from the two *S. lycopersicum* genomes, H1706, and YP, and very different from the wild species *S. chilense* and *S. galapagense*. Non-peak sequences of Gh13 were also nearly identical to *S. pimpinellifolium* sequences (REX fragments had 1 SNP, while the other two markers were identical) (Figures 4A, D, and E). The three markers tested in the SNP peak region, TG590, T0834, P6_051570 (Figure 2A), showed that the Gh13 sequence is different from the *S. lycopersicum* genomes, H1706, YP, and Purple Russian for TG590 and T0834 as well as for *S. pimpinellifolium* and *S. galapagense*. Other wild species tested for the chromosome 6 SNP peak region were two of the reported Gh13 pedigree parental lines of *S. habrochaites* (accessions LA1777 and LA0386) [42], and two other *Solanum chilense* accessions (LA2779 and LA1969) known to be sources of alleles of the *Ty-3* locus [21]. Phylogenetic analyses of the sequences for all three markers showed that Gh13 sequence was always closest to the two *S. chilense* accessions (Figure 4E) rather than the expected wild species S. *habrochaites*.

A similar approach was applied for chromosome 11, where we detected three candidate introgressed regions in the Gh13 genome (Figure 3A). The SNP plot of Gh13, *S. pimpinellifolium*, and the H1706 genome showed the Gh13 introgression regions overlap mostly with *S. pimpinellifolium* SNPs (Figure 5A). As expected, the seven markers tested in the three SNP peak regions showed that the Gh13 sequences had highest identity to *S. pimpinellifolium* (Figures 5D, and F). The six markers tested in the non-SNP-peak flanking regions all showed that Gh13 sequences were identical to the *S. lycopersicum* genomes H1706 and YP (Table 3, Figure 5E). Sequences for all thirteen markers on chromosome 11 were compared with those of two other wild tomato species. *S. chilense* sequences were mostly different than all the other genome sequences for all markers, and the *S. galapagense* sequence was intermediate between *S. lycopersicum* and *S. pimpinellifolium* (Figures 5D, E, and F; Table 3).

### SNP chip genotyping

The SolCAP SNP chip array containing 7,720 SNP markers [45] was used for genotyping Gh13 and HUJ-VF, a begomovirus-susceptible inbred. We defined regions having three or more polymorphic SNPs in 100 Kb as candidate introgressions, and found a total of 49 regions spanning 96.76 Mb with 968 polymorphic SNPs (Additional file 5: Table S3), compared with 171,711 SNPs spanning 49.42 Mb predicted with WGS. Of the 49 introgression-regions detected by the SolCAP chip, 25 have at least partial overlap with the Gh13 introgressions including, as expected, a full overlap with the predicted chromosome-6 introgression containing the *Ty-3* locus. The SolCAP introgressions that were not detected by WGS could be attributed to the comparison with two different susceptible lines (H1706 and HUJ-VF) that have different genome contents.

## Discussion

**Figure 6:**
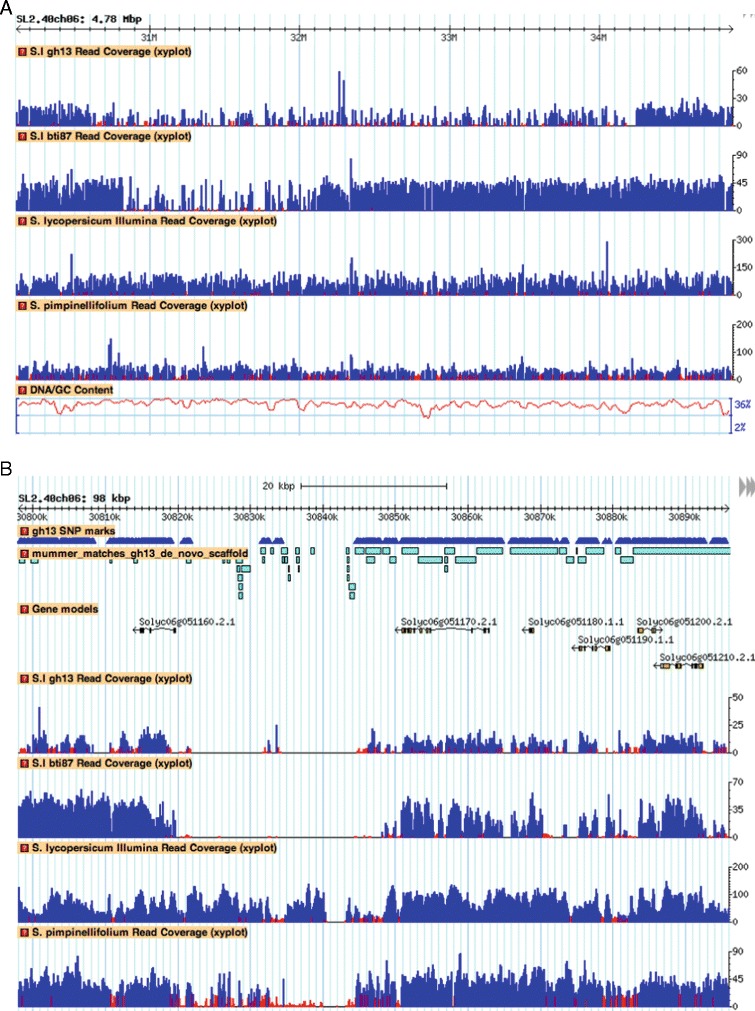
**Genome regions of the Ty-3 introgression in lines Gh13 and BTI-87. A)** Genome coverage plot of the chromosome 6 introgression (Gh13 and BTI-87). **B)** Zooming in an 80-Kb region from Figure 5A, spanning the Ty-3 region.

In this study, introgressions were detected and their origins inferred using whole-genome sequence analysis (re-sequencing), SNP calling, PCR sequencing, and phylogenetics. Two tomato inbreds (Gh13 and BTI-87) with alleles at the begomovirus resistance locus *Ty-3* were used to demonstrate that a known introgression for the *Ty-3* locus on chromosome 6 could be detected and boundaries determined (Figure 6A, and B). This re-sequencing strategy provides a wealth of polymorphism data (SNPs) between the reference genome and the re-sequenced lines Gh13 and BTI-87. To assess SNP regions, the chromosomes were divided into contiguous windows of 10 Kb. Plotting of the SNP frequency in each window, along the reference sequence, revealed regions of higher SNP density. These regions were tentatively labeled as introgressions. However, there were many smaller regions, from 40 Kb to a few hundred Kb in length, which showed high SNP density. These regions could represent smaller, ‘cryptic’ introgressions, or could be regions of high divergence due to other factors, such as transposon sequences. A total of 144 heretofore unknown putative introgressions, ranging in size from 50 Kb to more than 11 Mb, from different wild species were detected across the entire Gh13 genome, and 146 predicted introgressions in BTI-87 (ranging from 50 Kb to 42.87 Mb).

We detected, in both inbreds, chromosome-6 introgressions encompassing the Ty-3 locus. As the breeding pedigrees of these begomovirus-resistant lines are mostly unknown, yet both originate from a number of wild tomato species, we determined the origins of the introgressions by constructing phylogenetic trees based on sequencing of PCR fragments. Our results show that the introgressed regions in BTI-87 and in Gh13 cluster closely with *S. chilense*, identifying this wild species as the source for the *Ty-3* locus. Other notable introgressions were detected on chromosomes 4 and 11, where their origin is most likely *S. pimpinellifolium*. SNP peak regions that show high similarity between Gh13 and YP indicate introgressed region in H1706 from an unknown source, or from a different *S. pimpinellifolium* accession. The more than double the number of BTI-87 SNPs compared to Gh13 (Table 1; Additional file 2: Figure S2) is attributed to the large introgressions in chromosomes 4, 6 and 9. These results demonstrate that tomato breeding has resulted in numerous cryptic introgressions from various wild species. Current genome sequencing technologies, coupled with the available genomic resources, permit fast discovery of such candidate introgressions, could further assist in breeding programs, and facilitate the discovery of novel genetic variation and the study of gene function.

An important property of introgression detection is the ability to determine its boundaries accurately. The ability to detect the starting and ending nucleotide of the *S. chilense* introgression in chromosome 6 of Gh13 was tested by extracting the unique SNPs of *S. chilense* in the Gh13 genome by selecting only unique SNPs that do not occur in the other tested genomes, having a coverage greater than 10× and allele frequency greater than 90%. This analysis yielded 4,931 unique *S. chilense* SNP positions in the Gh13 genome, with 148 SNPs in the 30.6- to 34.22-Mb chromosome 6 region of the predicted *S. chilense* introgression. The first SNP position within this region is at nucleotide 30,620,481, and the last is at nucleotide 34,051,365. This analysis should be repeated with the fully sequenced reference genome of *S. chilense* and other wild parental lines for delineating the accurate introgressions throughout the genome. The SolCAP SNP chip gave similar results for the *Ty-3* introgression (30,623,784 to 33,972,992 nucleotides); however, only 29 SNPs were polymorphic, compared to more than 35,000 SNPs detected with WGS, thereby providing a greater breadth of data related to the introgression content.

The *Ty-1* and *Ty-3* loci were recently mapped to the same region of chromosome 6 [21], which is within the introgression for chromosome 6 for both Gh13 and BTI-87. Mapping the *Ty-1* and *Ty-3* loci was time-consuming and required large mapping populations over many generations of selection [21]. With re-sequencing and SNP analysis, it is possible to facilitate fine-mapping and eventually cloning of a target gene, since putative introgressions from wild species can be easily detected and possibly narrow the genomic region to be screened.

## Conclusions

We utilized the H1706 reference genome and other genome sequences from *S. pimpinellifolium*, *S. chilense*, and YP, to detect introgressions in two begomovirus-resistant inbreds and identify the origin of some of these introgressions. The discovered introgressions vary greatly in size, location, and content, and our analysis with the heirloom line YP shows many of the introgressions are in the H1706 genome, which is known to have *S. pimpinellifolium* in its pedigree. These findings emphasize the need for additional genomic sequences of tomato wild species, which can be used to identify the origin of tomato introgressions, and study genome sequences that may not exist in the H1706 genome [46]. In addition, approaches outlined here can be used to develop SNP markers for specific regions and to determine the boundaries for introgressions. Our approach, in this report, represents a proof of concept that can readily be applied to other species with available reference genomes.

## Methods

### Plant material

*Solanum lycopersicum* inbred Gh13 was derived from the TYLCV-resistant germplasm FAVI 9 [42] by multiple generation selection of single begomovirus-resistant plants in the field in Sanarate, Guatemala [41,46]. Disease resistance genes in Gh13 were detected by SNP analysis by AgBiotech, Inc. and results were: homozygous for the begomovirus-resistance locus *Ty-3* on chromosome 6; homozygous for *Ve* on chromosome 9; heterozygous for *I2* on chromosome 11, susceptible for *Mi*, *Sw5*, *Ty2*, *Ph3*, *Tm2a*, and *Pto*. Molecular scanning by sequencing PCR fragments showed that Gh13 had an introgression on chromosome 6 from 20 to 32 cM (C. Martin and D.P. Maxwell, personal communication), which corresponds to the location of the *Ty-3* locus [47,48]. Gh13 was used in several research projects to determine the effectiveness of the *Ty-3* locus in conferring resistance to begomoviruses [40,49].

The proprietary begomovirus-resistant *S. lycopersicum* inbred, BTI-87, was obtained from the commercial seed company Semillas Tropicales, S.A. The source of begomovirus resistance in BTI-87 was from the inbred line Gc171, which is known to have the *Ty-3a* and *Ty-4* resistance loci on chromosome 6 and chromosome 3, respectively [47,50]. These resistant loci were introgressed from *S. chilense* LA1932 [43]. Disease resistance genes in BTI-87 were detected by SNP analysis by AgBiotech, Inc. and results were: homozygous for the begomovirus-resistance locus *Ty-3 or Ty-3a* on chromosome 6; heterozygous for *Mi* on chromosome 6; homozygous for the gene *Tm2a* on chromosome 9; and susceptible for *I2* and *Sw5*.

Seeds of accessions *S. habrochaites* LA0386 and LA1777, *S. chilense* LA1932, LA1969, and LA2779, and *S. galapagense* LA0436 were obtained from the Tomato Genetics Resource Center at UC Davis (http://tgrc.ucdavis.edu).

Seeds of *S. lycopersicum* H1706 (LA4345) and YP were provided by Gregory Martin, Boyce Thompson Institute for Plant Research (BTI). *S. lycopersicum* Purple Russian seeds were available from the laboratory of Douglas Maxwell, University of Wisconsin-Madison. The SNP assay for resistance loci by AgBiotech, Inc. showed that the *S. lycopersicum* lines, H1706, YP, and Purple Russian, had susceptible loci for *Ty-3*, *Mi*, *I2*, *Sw5*, and *Tm2a*.

### DNA extraction

Gh13 seedlings were grown at the University of Wisconsin-Madison. DNA was extracted using CTAB method [51], yielding about 500 ng/ul of genomic DNA for whole-genome sequencing.

About 20 seedlings of tomato line BTI-87 were grown in a greenhouse under standard conditions (22°C, 14 h light) at Boyce Thompson Institute for Plant Research. Young leaves of 4- week-old seedlings were collected for DNA extraction using CsCl gradient as described previously [52]. Plants of Purple Russian, LA0386, LA1777, LA1932, LA1969, LA2779, and H1706 (LA4345) were grown under the same conditions as BTI-87 and young leaf tissue was collected and DNA extracted with CTAB protocol.

### Genome sequencing

Paired-end (PE) libraries of Gh13, BTI-87, and *S. chilense* LA1932 were generated and sequenced on Illumina HiSeq 2000 machine at the Weill-Cornell Genomics Core Facility, New York, NY. Each PE library had an insert size of 300 bp. The reference genome for *S. lycopersicum* H1706 used is from the international tomato genome project, version SL2.40 (http://solgenomics.net/organism/Solanum_lycopersicum/genome). Dr. Zach Lippman, at the Cold Spring Harbor Laboratory, sequenced the *S. pimpinellifolium* accession, LA1589, [24]. *S. galapagense* accession LA0436 and the *S. lycopersicum* heirloom line YP sequences were obtained from a previous study at BTI [37].

### Genome assembly

Illumina reads were inspected for quality using FastQC and rechecked after cleaning. Cleaning was performed with fastq-mcf (http://www.bioinformatics.babraham.ac.uk/projects/fastqc/). Reads were mapped to the *S. lycopersicum* H1706 reference assembly version 2.40 using BWA [53] with default parameters. Duplicate reads as well as reads with a mapping quality less than 30 were removed for variation analysis with Picard (http://picard.sourceforge.net) and Samtools (http://samtools.sourceforge.net/) [54], respectively. SNPs and indels were detected using Samtools mpileup (http://samtools.sourceforge.net/mpileup.shtml).

Whole genome *de novo* assemblies of Gh13 and BTI-87 were created using SOAPdenovo version 1.05 (http://soap.genomics.org.cn/) [55]. Assemblies were produced using a kmer range between 25 and 63. Scripts supplied with the SOAPdenovo package were used for error correction and gap filling of the scaffolds. De novo reads were mapped to the reference H1706 genome to increase coverage in regions with poor mapping from the BWA-aligned sequences.

For determining exact *S. chilense* introgression breakpoints in Gh13, variants of accession LA1932 were called using VarScan2 [56] and unique LA1932 SNPs in the Gh13 genomes were extracted using custom Perl scripts (https://github.com/nmenda/GenomeTools).

### SNP plots

SNPs of *S. pimpinellifolium*, Gh13, and BTI87 that were called in reference to H1706 were compared to each other, and labeled ‘unique’ or ‘common’. SNPs for each group were then aggregated into bins of 10 Kb using a custom Perl script (https://github.com/nmenda/GenomeTools). SNP density for each comparison was plotted along every *S. lycopersicum* ‘Heinz’ chromosome using R statistics (http://www.R-project.org).

### Introgression detection

Introgressions were defined as SNP-peaks having at least 10 SNPs per 10 Kb window, with minimum size of 50 Kb, and up to 40 Kb of continuous gaps. Minimum size was chosen for capturing small introgressions, and the gaps were introduced to offset the significant decrease in genome coverage in introgressed regions due to the difficulty to map those regions to the reference H1706 genome. The minimum number of SNPs per window was selected based on the hypothesis that having no introgressions means the average number of SNPs per 10 Kb window in the entire genome will be similar to this number in non-peak regions. If introgressions can be defined as having significantly higher number of SNPs in peak-regions and lower number of SNPs in non-peak regions, then the average number of SNPs per window in the entire genome should be higher than the number of SNPs in the non-peak regions. We tested introgressions using minimum number of 3, 5, 10, 15, or 20 SNPs per 10 Kb, extracting for each condition the SNP-peak and non-peak regions, and comparing the average number of SNPs in 10 Kb windows in the non-peak regions to that number in the entire genome of Gh13, and comparing each pair using Student’s t-test [57,58].

### PCR and Sanger sequencing

PCR primers were developed for regions of interest based on previous markers and genic regions. PCR products were generated from *S. chilense*, *S. habrochaites*, and *S. lycopersicum* (lines Gh13, and Purple Russian). PCR was performed at 55 degrees Celsius, 32 amplification cycles, 60 seconds extension step. All designed primers are listed in Table 3. PCR products were cleaned with Qiagen QIAquick PCR Purification Kit, and sent for Sanger sequencing to the Life Science Core Laboratory Center at Cornell University (Ithaca, NY) or to the University of Wisconsin-Madison Biotechnology Center. Sequences from *S. lycopersicum* H1706 and YP, the inbred BTI-87, *S. pimpinellifolium*, and *S. galapagense* were extracted from their genome assemblies by best BLAST match of primer pairs.

### Phylogenetic trees

Putative orthologous sequences for regions of interest were obtained from draft genome assemblies by using *S. lycopersicum* H1706 sequence selecting the top BLAST hit followed by reciprocal BLAST back to *S. lycopersicum* H1706. Sequences from Gh13, BTI-87, *S. lycopersicum* H1706, YP and Purple Russian, *S. pimpinellifolium*, *S. galapagense*, *S. chilense*, and *S. habrochaites* when available, were aligned using ClustalW [59] with default settings. Alignments were inspected to ensure accuracy. Mega5 was used to construct maximum likelihood trees using 500 bootstrap replicates and the Tamura-Nei substitution model [60]. FigTree (http://tree.bio.ed.ac.uk/software/figtree/) was used for drawing the gene tree figures. All trees were submitted to TreeBase http://purl.org/phylo/treebase/phylows/study/TB2:S16453.

### SNP array genotyping

Lines Gh13 and a begomovirus-susceptible inbred, HUJ-VF that lacked the *Ty-3* locus, were genotyped using a tomato array with 7,720 SNPs as implemented in the Infinium assay (Illumina Inc., San Diego, CA, USA). HUJ-VF, a processing type tomato, was provided by Dr. Favi Vidavsky, Hebrew University of Jerusalem. For each accession, genomic DNA was isolated from fresh, young leaf tissue using a Qiagen DNeasy kit (Qiagen, USA) at the University of Wisconsin-Madison. Double-stranded DNA concentrations were quantified using the PicoGreen assay (Life Technologies Corp., Grand Island, NY, USA) and normalized to 50 ng/ul with 10 mM Tris–HCl pH 8.0, 1 mM EDTA. Genotyping was conducted with 250 ng of DNA per accession following the manufacturer’s protocol for the Infinium assay. For SNP calls, the resulting intensity data was loaded in GenomeStudio version 1.7.4 (Illumina Inc., San Diego, CA, USA). In order to determine SNP genotype, the automated cluster algorithm was first used to generate initial SNP calls. Clustering for every SNP was determined using the SolCAP cluster file [45].

### Availability of supporting data

The genomes of lines Gh13 and BTI-87 are available to browse, BLAST, and download at the Sol Genomics Network website (http://solgenomics.net/organism/Solanum_lycopersicum/inbred_genomes). Sequences of PCR products and primers designed and sequences in this work are available from the NCBI GenBank nucleotide database, accession numbers KF887310–KF887341.

Custom perl scripts are available from GitHub https://github.com/nmenda/GenomeTools.

## Additional files

Additional file 1: Figure S1. Gh13 SNP density and coverage plots. X axes are positions in bp, Y axes are number of SNPs, and negative Y axes are genome coverage. Introgression regions are highlighted in red. Additional file 2: Figure S2. BTI-87 SNP density and coverage plots. X axes are positions in bp, Y axes are number of SNPs, and negative Y axes are genome coverage. Introgression regions are highlighted in red. Additional file 3: Table S1. Introgressions in Gh13 by 10 Kb windows. Overlapping SNPs of YP, *S. pimpinellifolium*, BTI-87, *S. chilense* LA1932, and their overlapping SNPs with Gh13. Additional file 4: Table S2. Summary of introgressions in BTI-87 by 10 Kb windows. Additional file 5: Table S3. Gh13 introgressions summary and SolCAP introgression regions.

## Abbreviations

SNP: Single nucleotide polymorphism
WGS: Whole genome sequencing
YP: Yellow pear
H1706: Heinz 1706
PR: Purple Russian
