# Supplementary material for: Analysis of wild-species introgressions in tomato inbreds uncovers ancestral origins
Source: BMC Plant Biol. 2014 Oct 28;14:287. doi: 10.1186/s12870-014-0287-2 (PMC4219026; doi:10.1186/s12870-014-0287-2)
Supplement: Additional file 1: Figure S1. — Gh13 SNP density and coverage plots. X axes are positions in bp, Y axes are number of SNPs, and negative Y axes are genome coverage. Introgression regions are highlighted in red. [file 12870_2014_287_MOESM1_ESM.pdf]

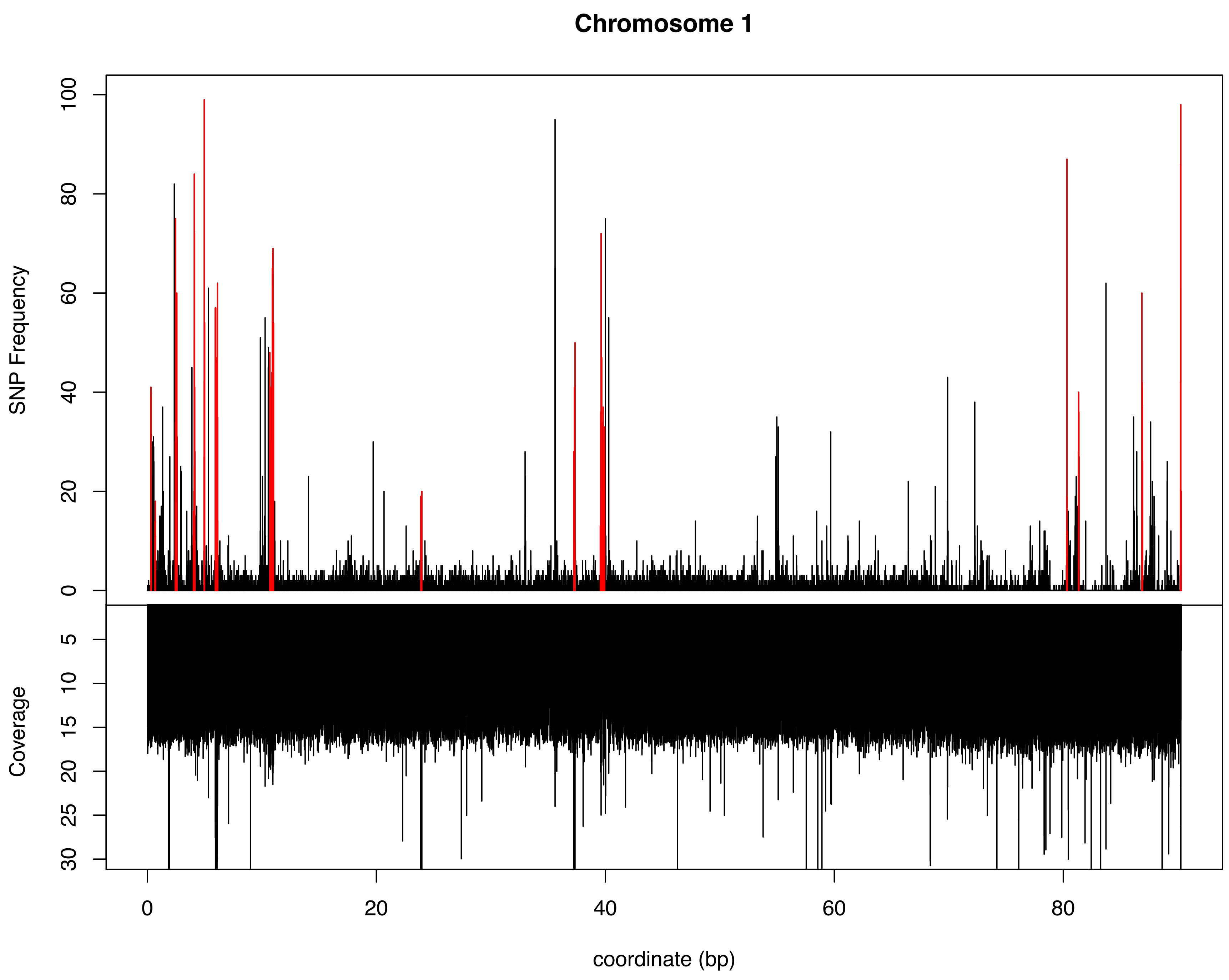

Chromosome 1
SNP Frequency
0
20
40
60
80
100
Coverage
5
10
15
20
25
30
0
20
40
60
80
coordinate (bp)

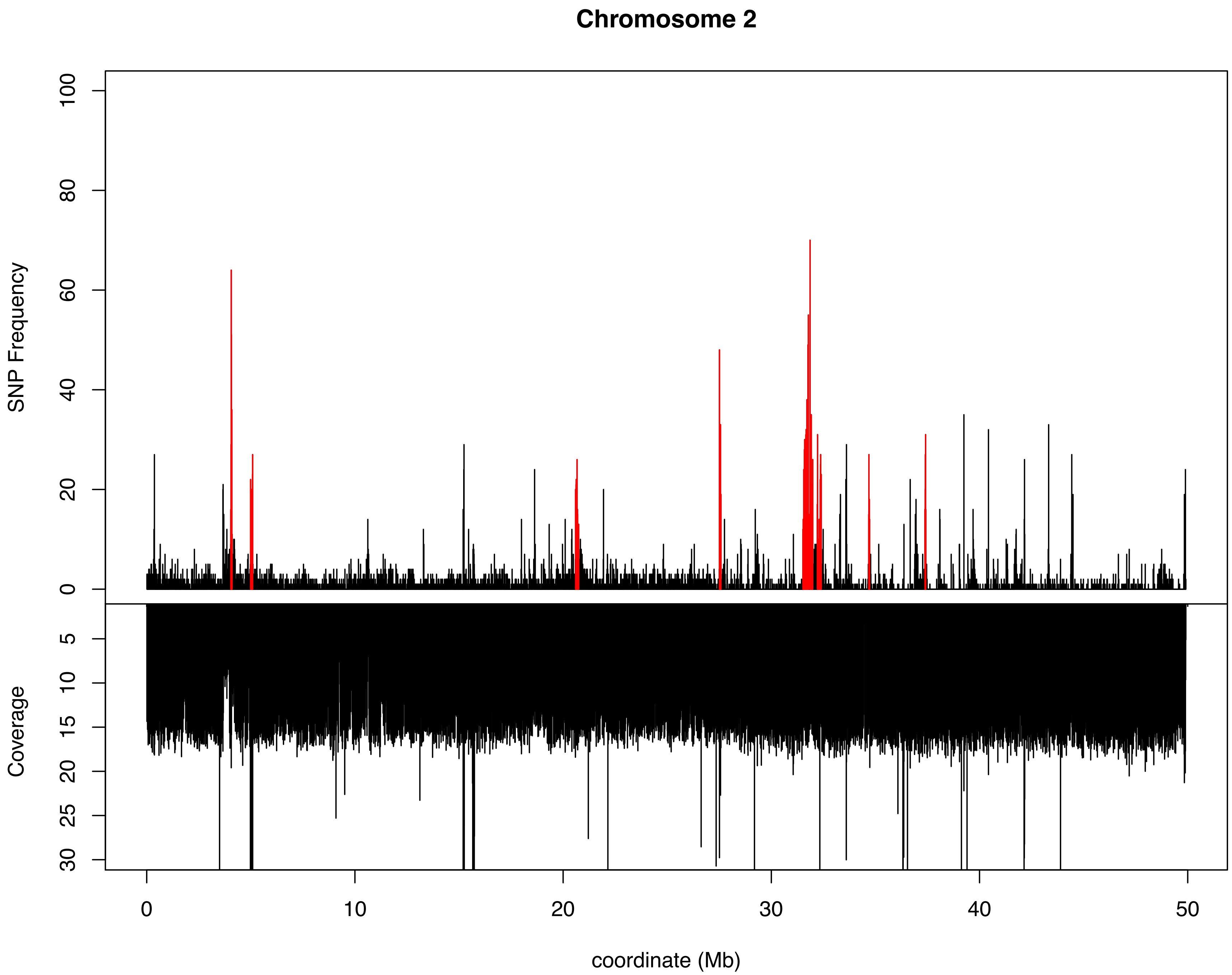

Chromosome 2
SNP Frequency
Coverage
coordinate (Mb)
0
20
40
60
80
100
5
10
15
20
25
30
0
10
20
30
40
50

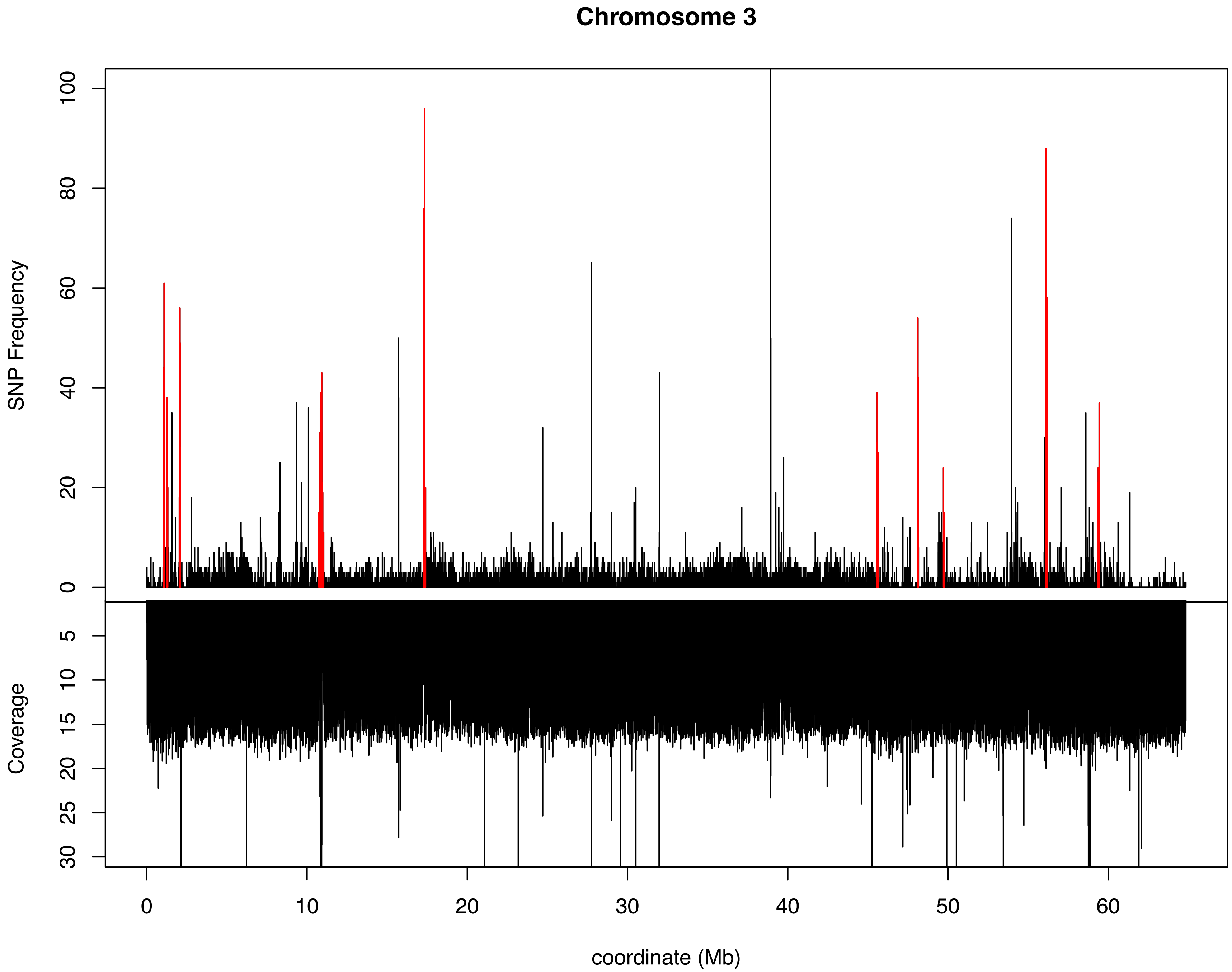
Chromosome 3
SNP Frequency
0
20
40
60
80
100
Coverage
5
10
15
20
25
30
0
10
20
30
40
50
60
coordinate (Mb)

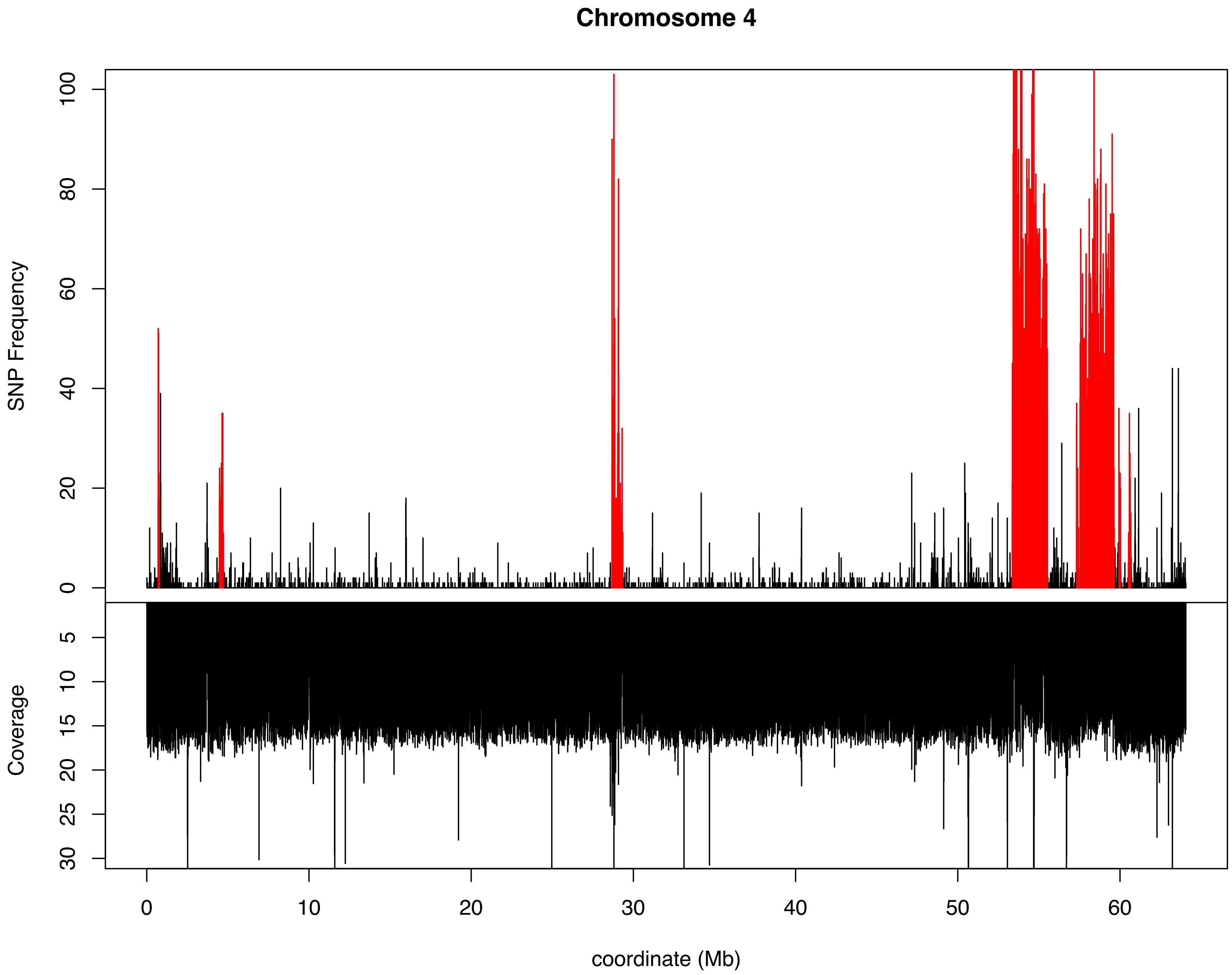

Chromosome 4
SNP Frequency
0
20
40
60
80
100
Coverage
5
10
15
20
25
30
0
10
20
30
40
50
60
coordinate (Mb)

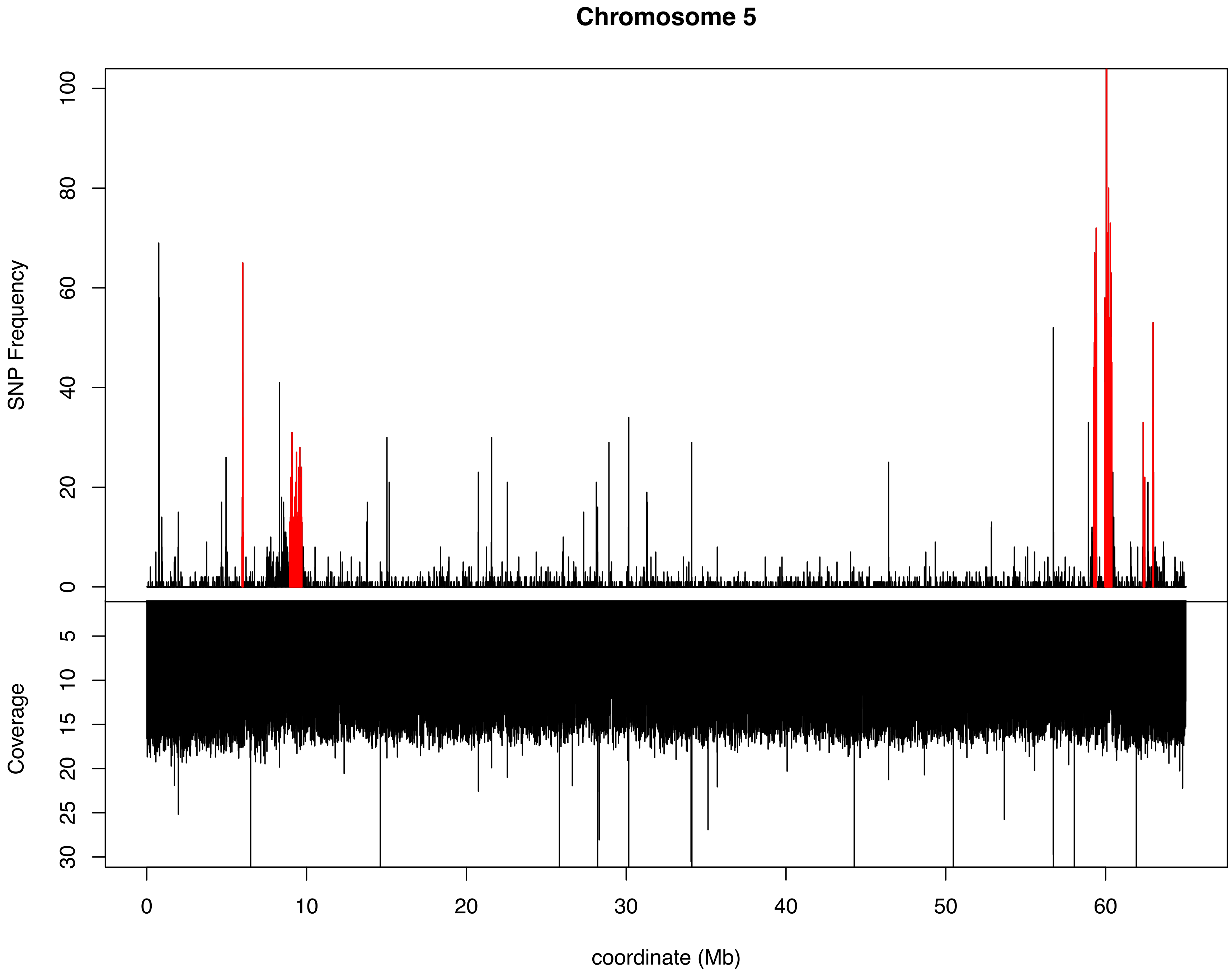
Chromosome 5
SNP Frequency
0
20
40
60
80
100
Coverage
5
10
15
20
25
30
0
10
20
30
40
50
60
coordinate (Mb)

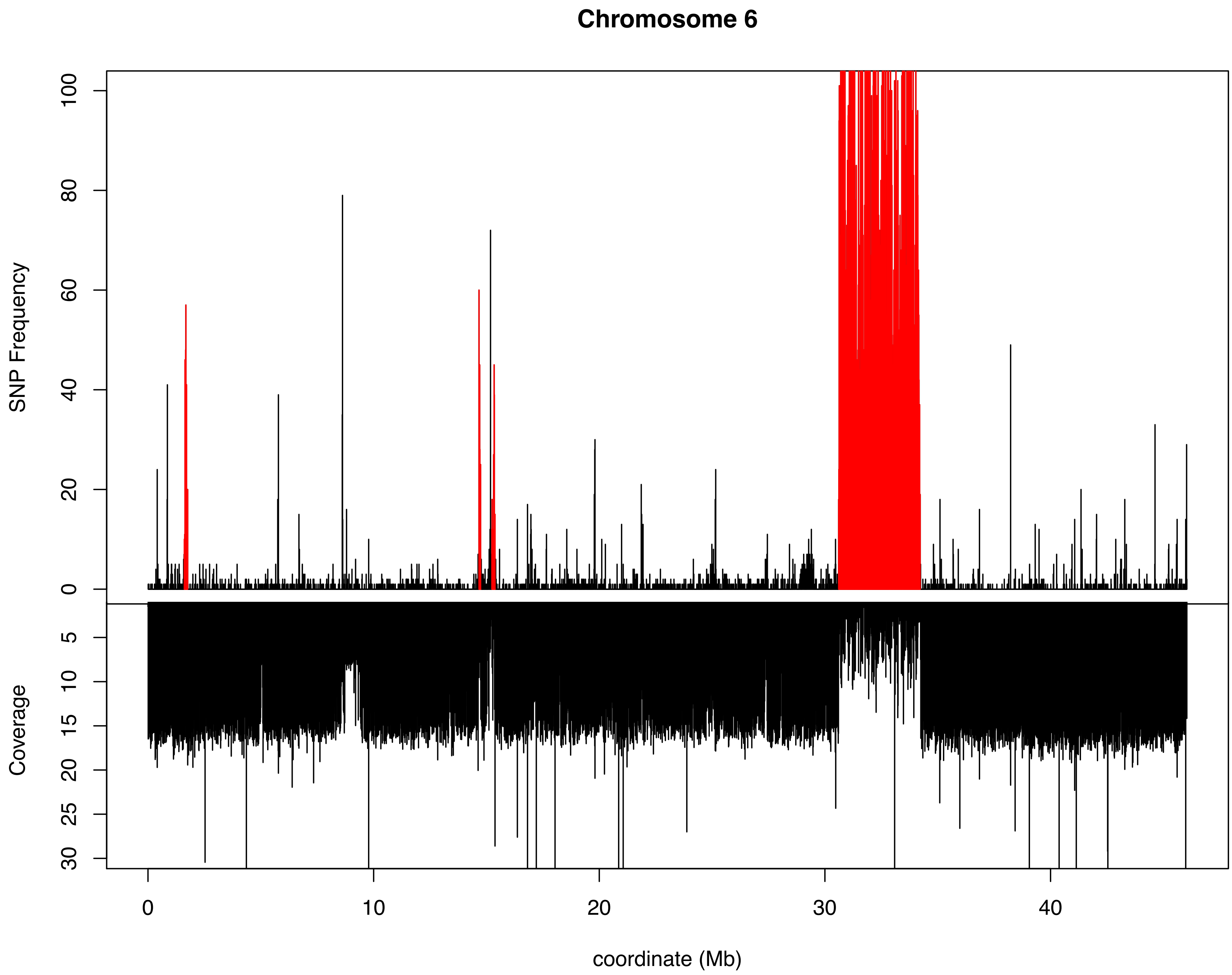
Chromosome 6
SNP Frequency
0
20
40
60
80
100
Coverage
5
10
15
20
25
30
0
10
20
30
40
coordinate (Mb)

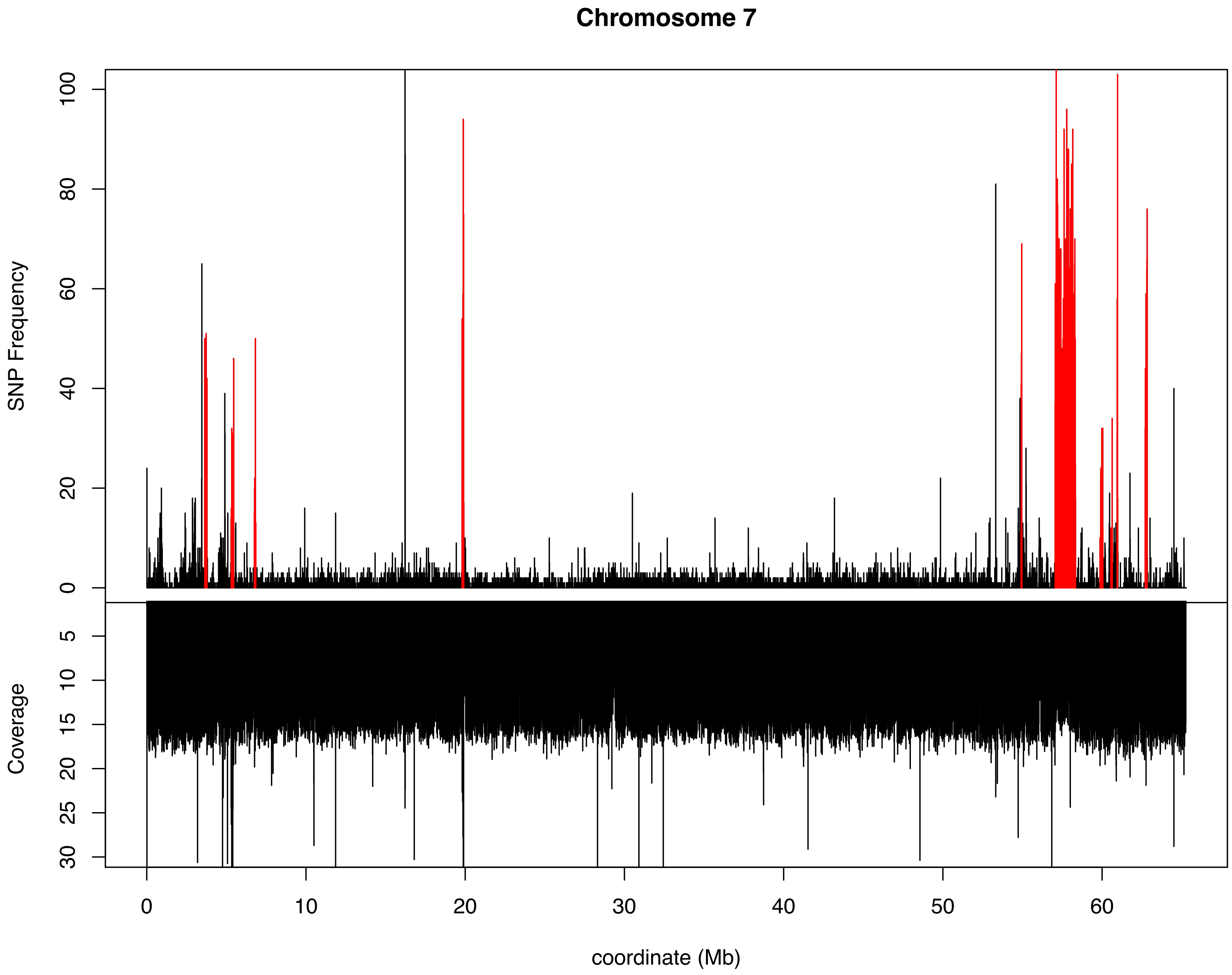
Chromosome 7
SNP Frequency
0
20
40
60
80
100
Coverage
5
10
15
20
25
30
0
10
20
30
40
50
60
coordinate (Mb)

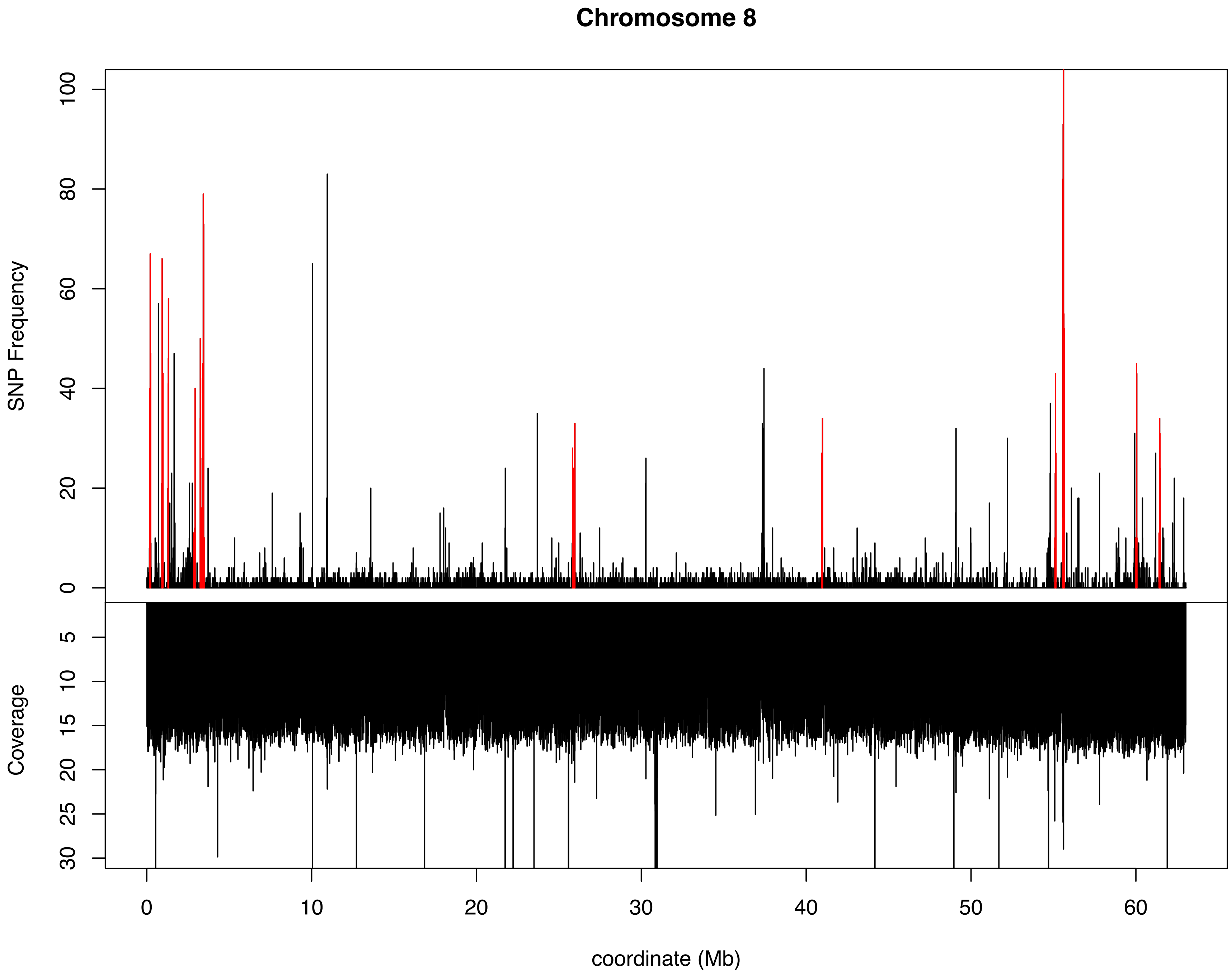

Chromosome 8
SNP Frequency
0
20
40
60
80
100
Coverage
5
10
15
20
25
30
0
10
20
30
40
50
60
coordinate (Mb)

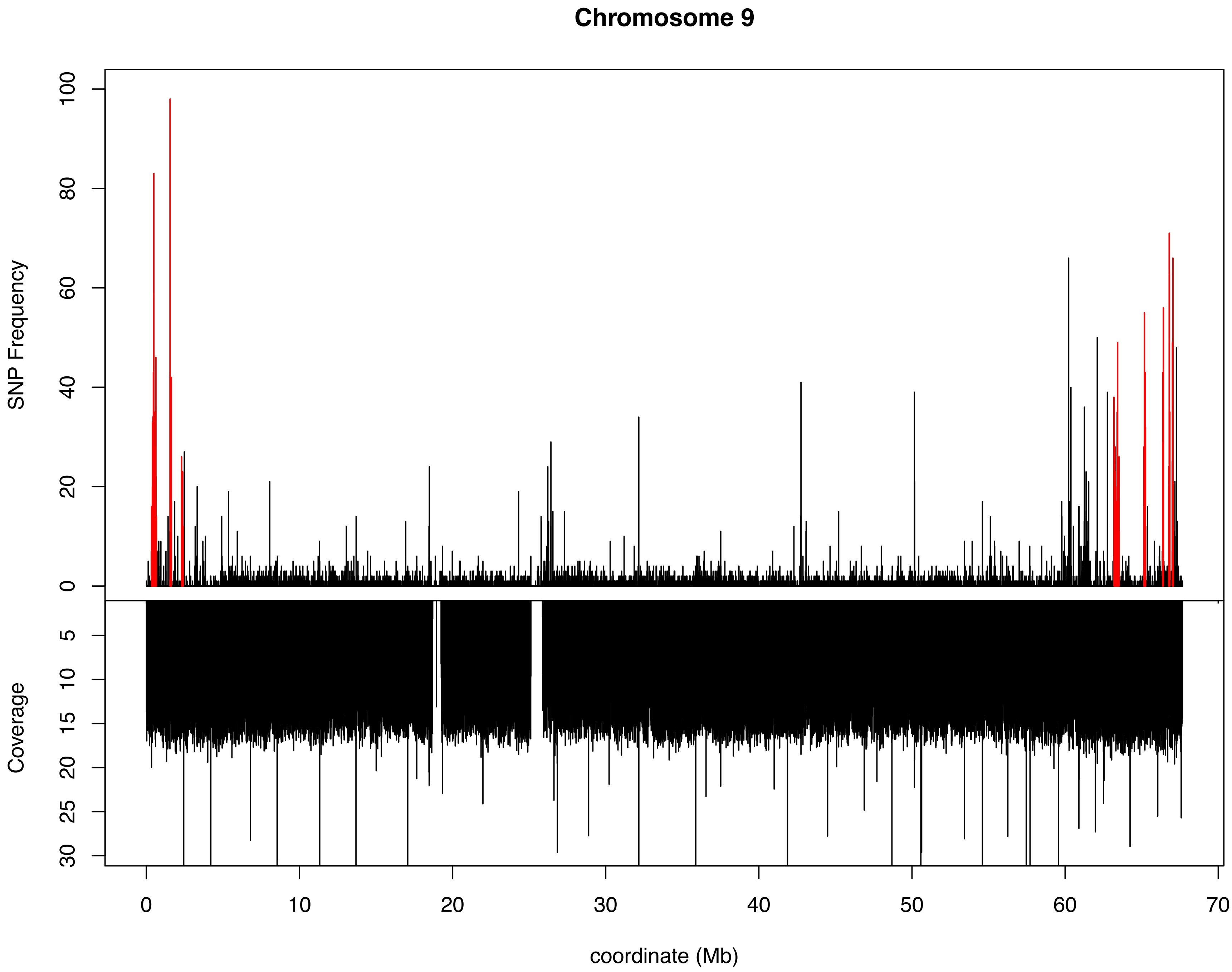
Chromosome 9
SNP Frequency
0
20
40
60
80
100
Coverage
5
10
15
20
25
30
0
10
20
30
40
50
60
70
coordinate (Mb)

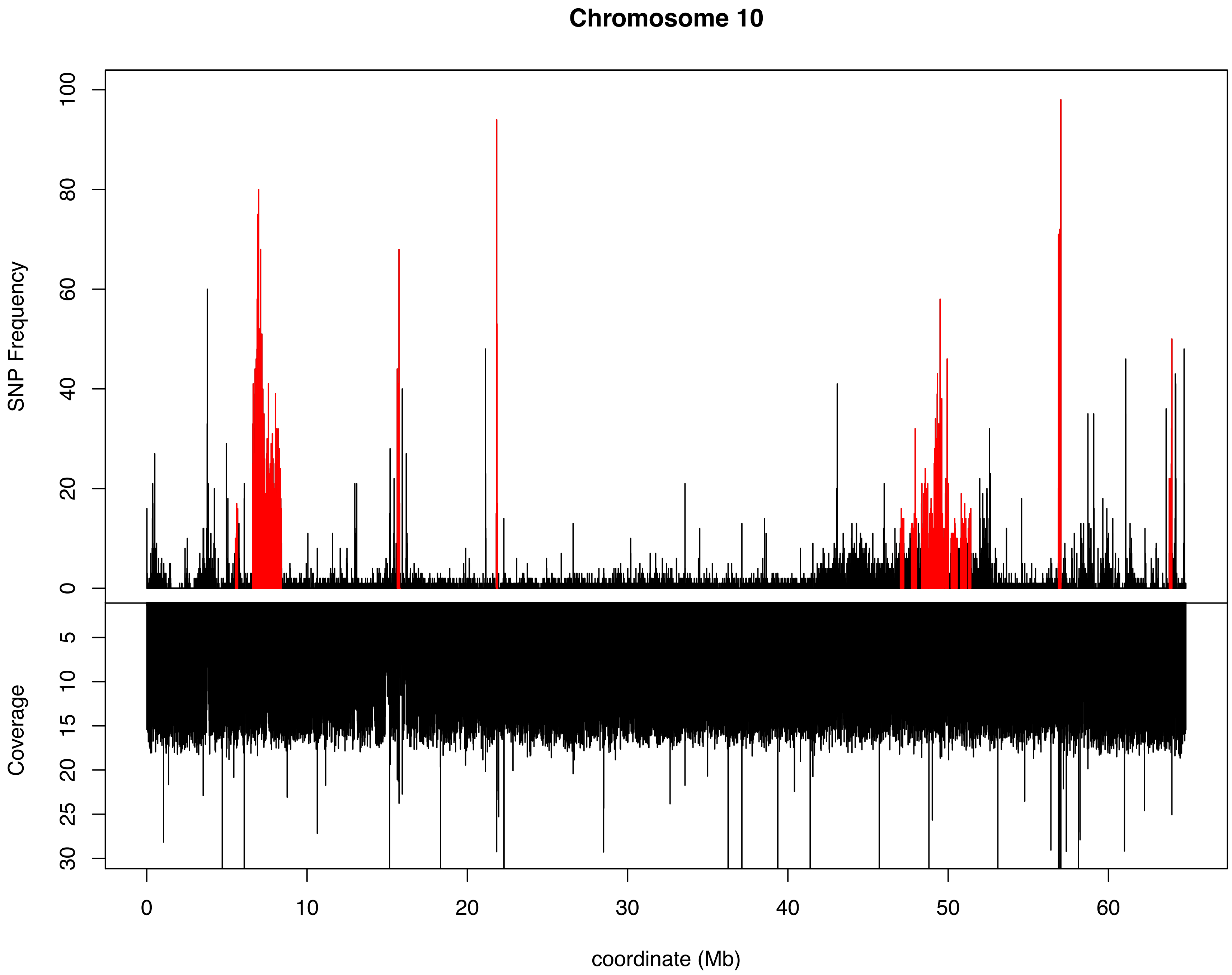

Chromosome 10
SNP Frequency
0
20
40
60
80
100
Coverage
5
10
15
20
25
30
0
10
20
30
40
50
60
coordinate (Mb)

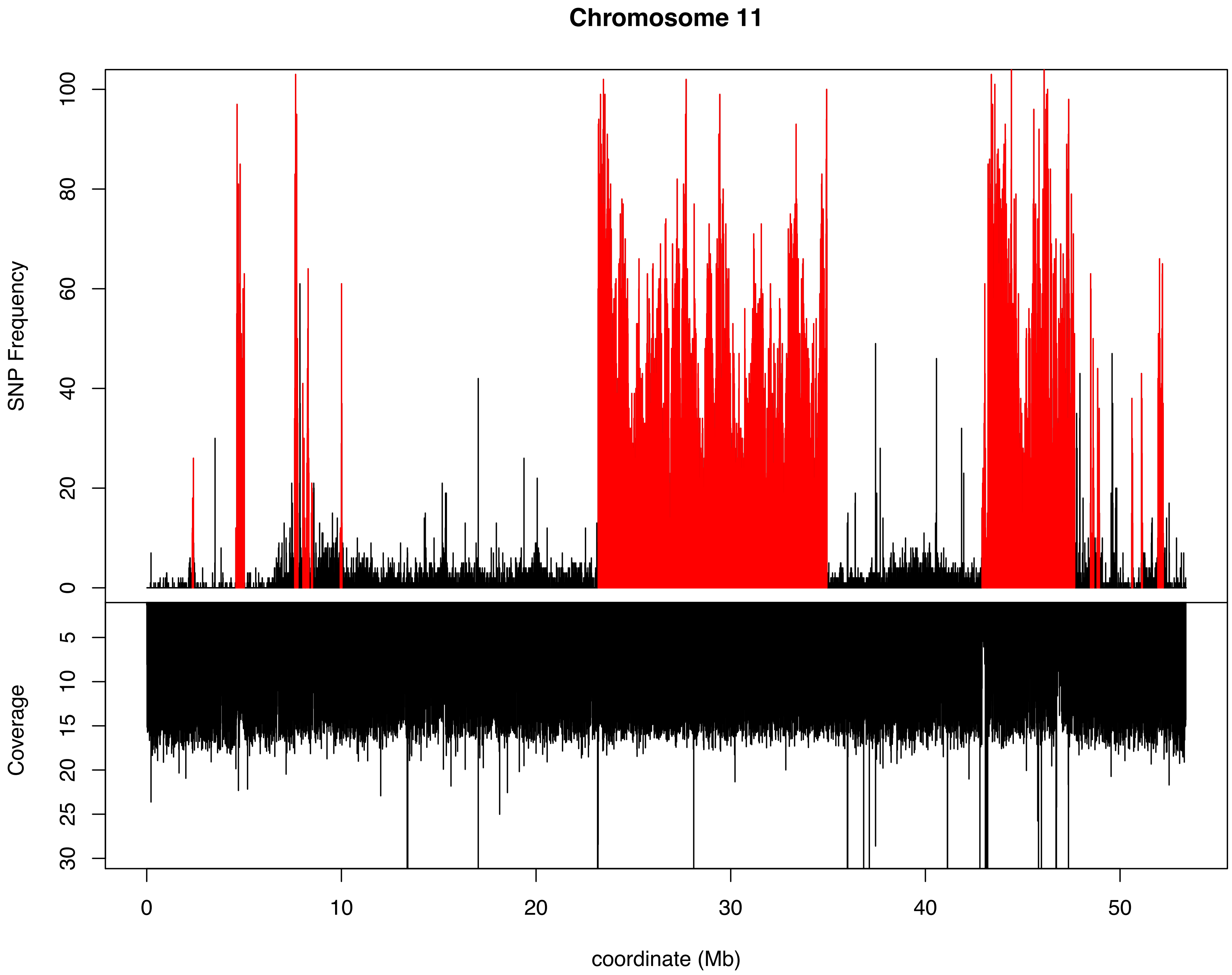

Chromosome 11
SNP Frequency
Coverage
coordinate (Mb)
0
20
40
60
80
100
5
10
15
20
25
30
0
10
20
30
40
50

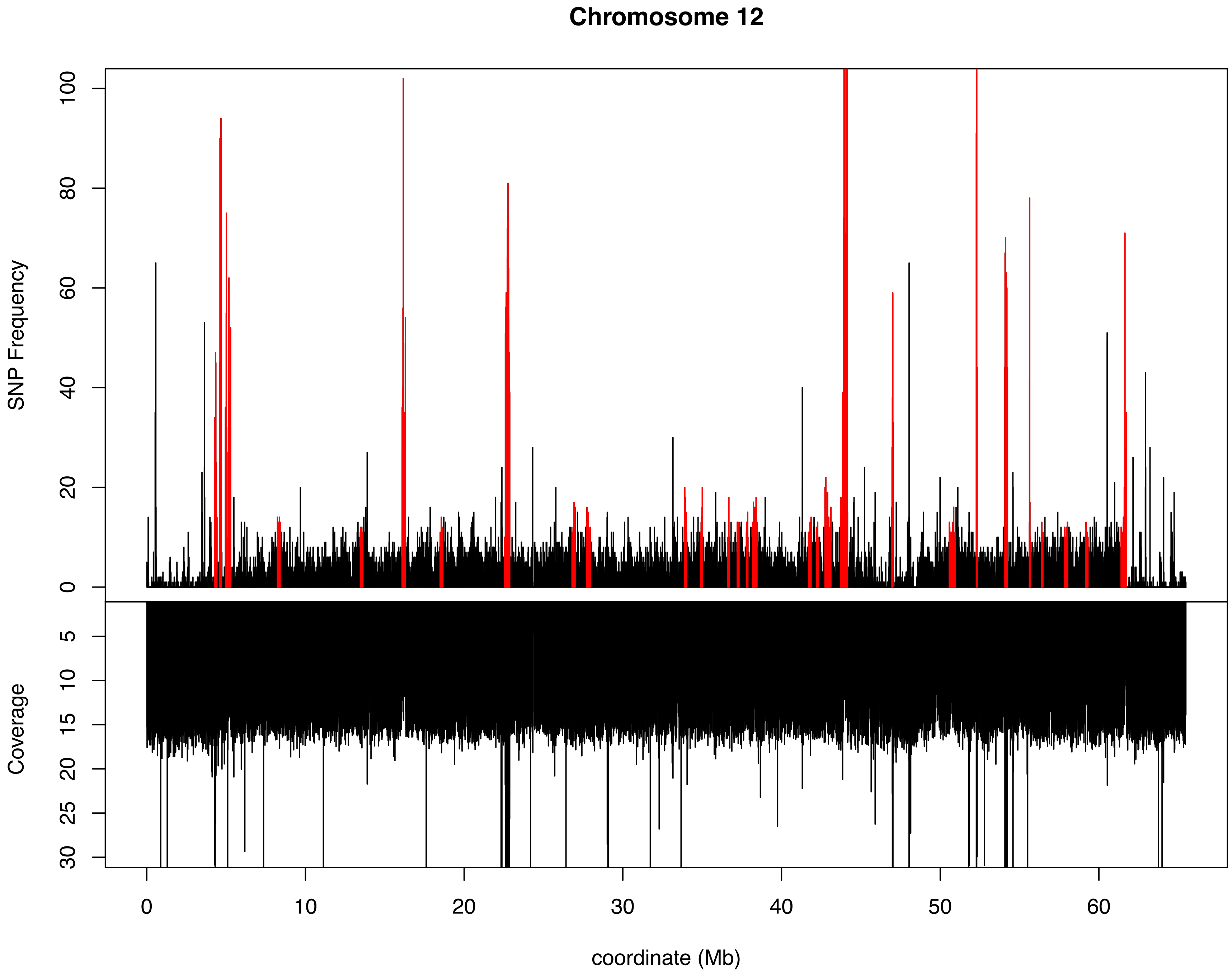

Chromosome 12
SNP Frequency
Coverage
0
20
40
60
80
100
5
10
15
20
25
30
0
10
20
30
40
50
60
coordinate (Mb)
