# Supplementary material for: Analysis of wild-species introgressions in tomato inbreds uncovers ancestral origins
Source: BMC Plant Biol. 2014 Oct 28;14:287. doi: 10.1186/s12870-014-0287-2 (PMC4219026; doi:10.1186/s12870-014-0287-2)
Supplement: Additional file 2: Figure S2. — BTI-87 SNP density and coverage plots. X axes are positions in bp, Y axes are number of SNPs, and negative Y axes are genome coverage. Introgression regions are highlighted in red. [file 12870_2014_287_MOESM2_ESM.pdf]

Chromosome 1

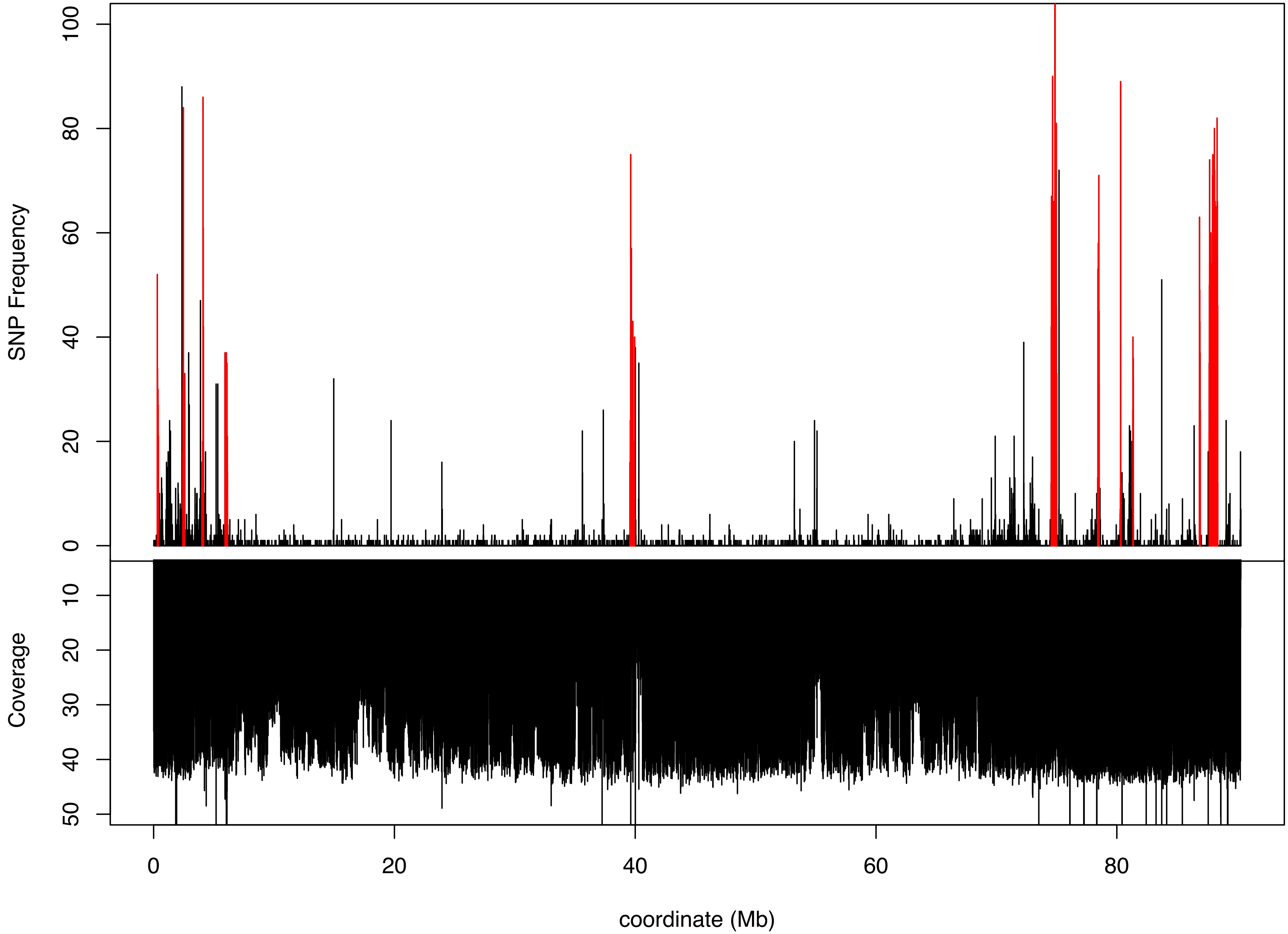

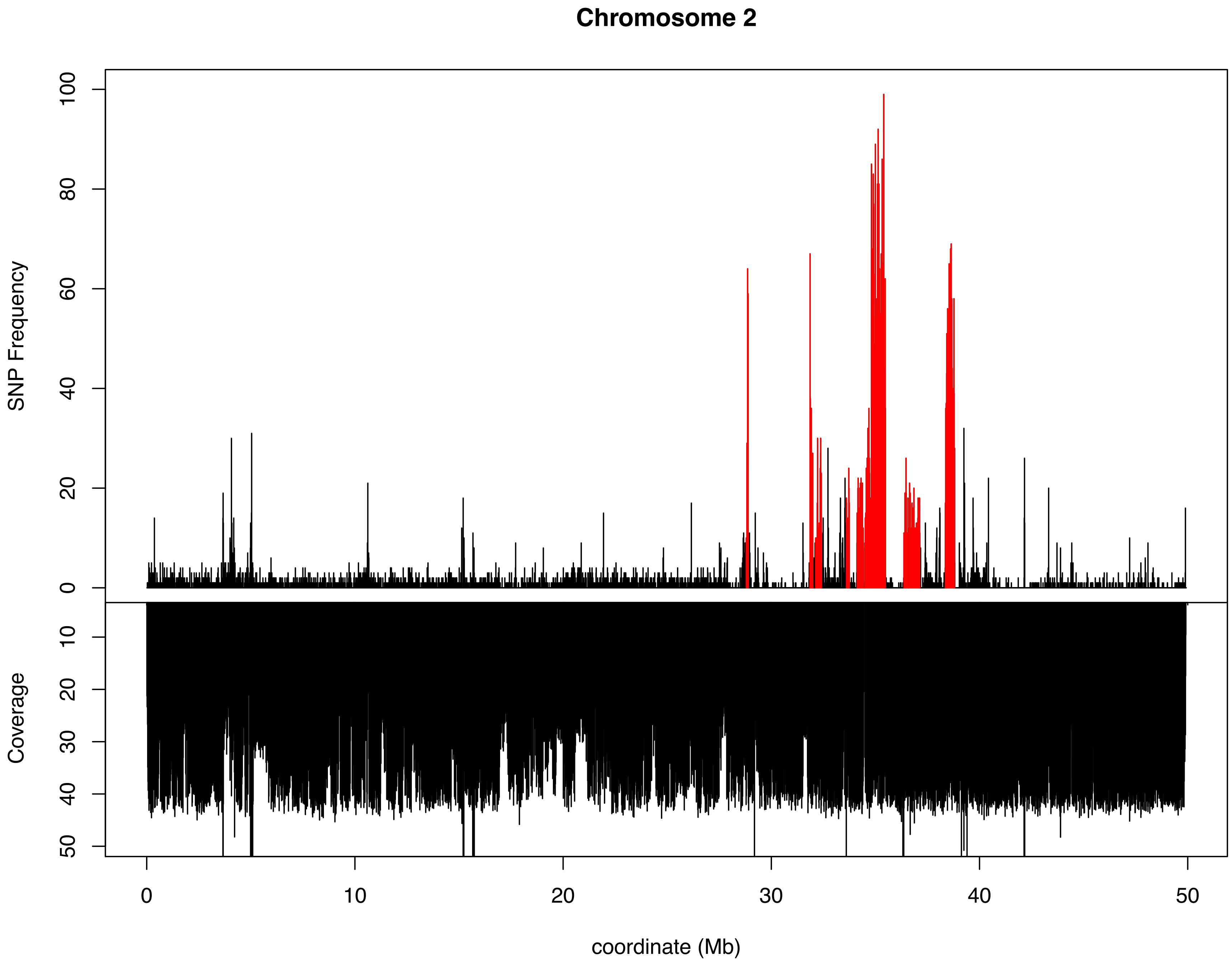

Chromosome 2
SNP Frequency
Coverage
coordinate (Mb)
0
20
40
60
80
100
10
20
30
40
50
0
10
20
30
40
50

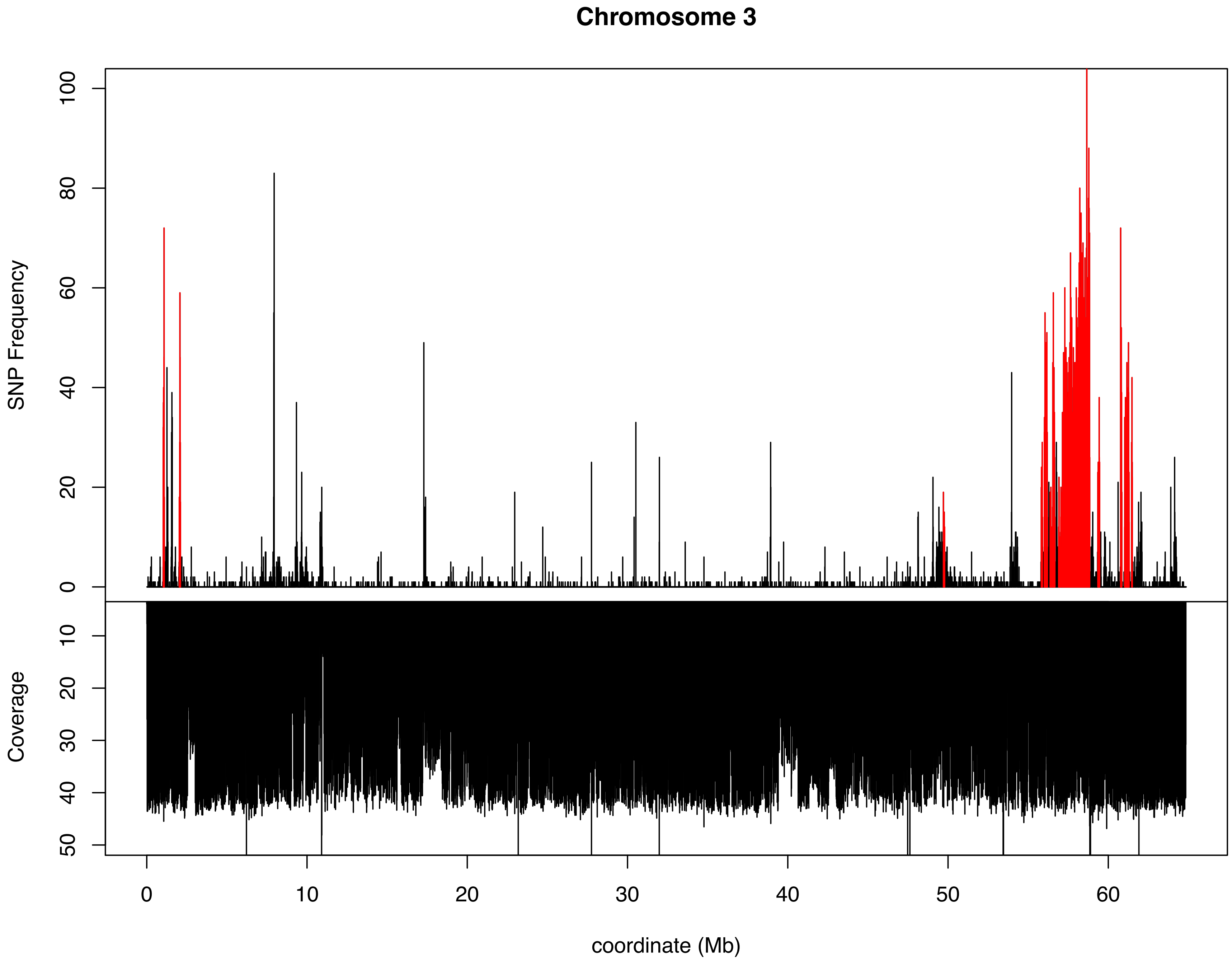

Chromosome 3
SNP Frequency
0
20
40
60
80
100
Coverage
10
20
30
40
50
0
10
20
30
40
50
60
coordinate (Mb)

Chromosome 4

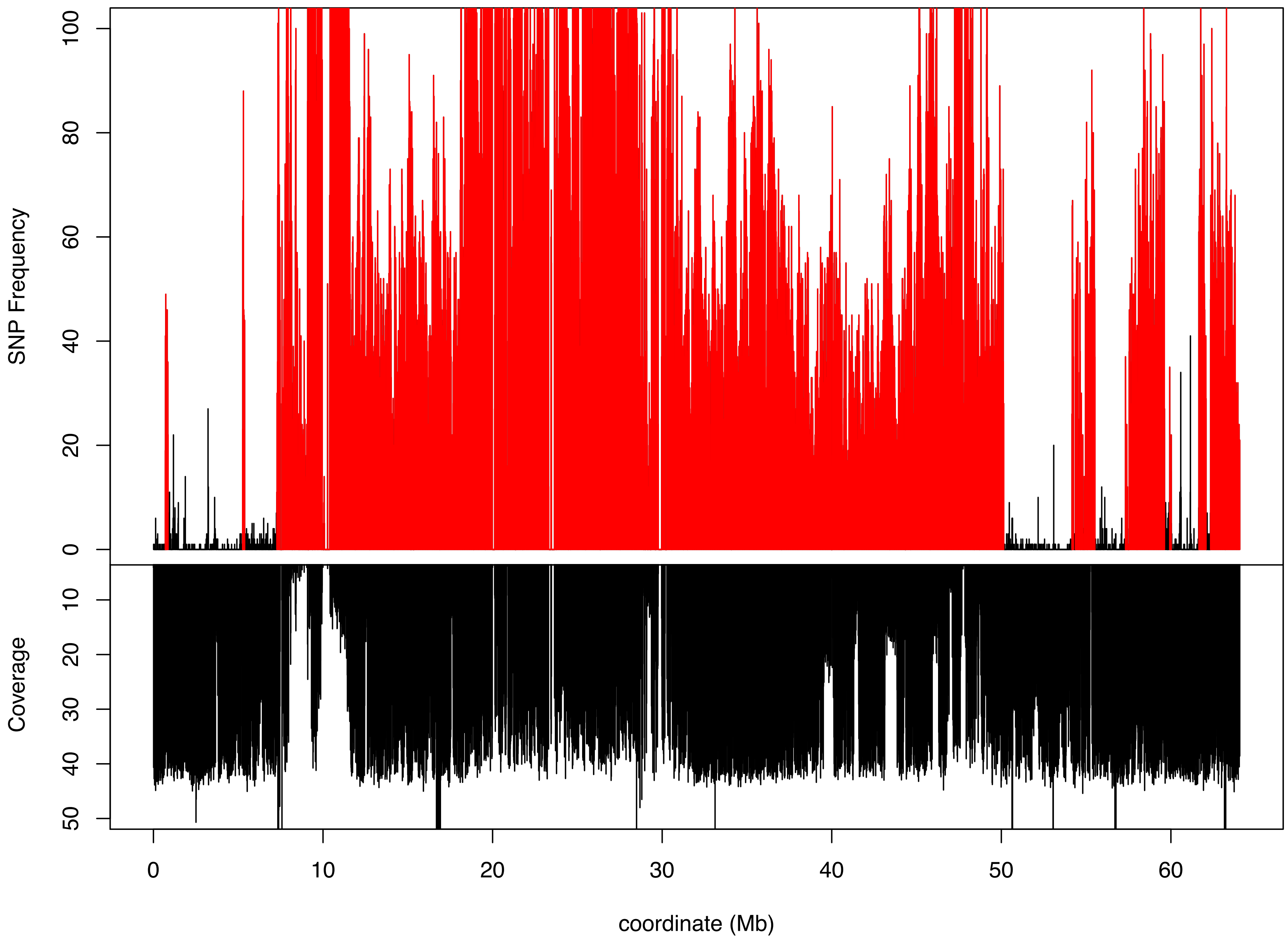

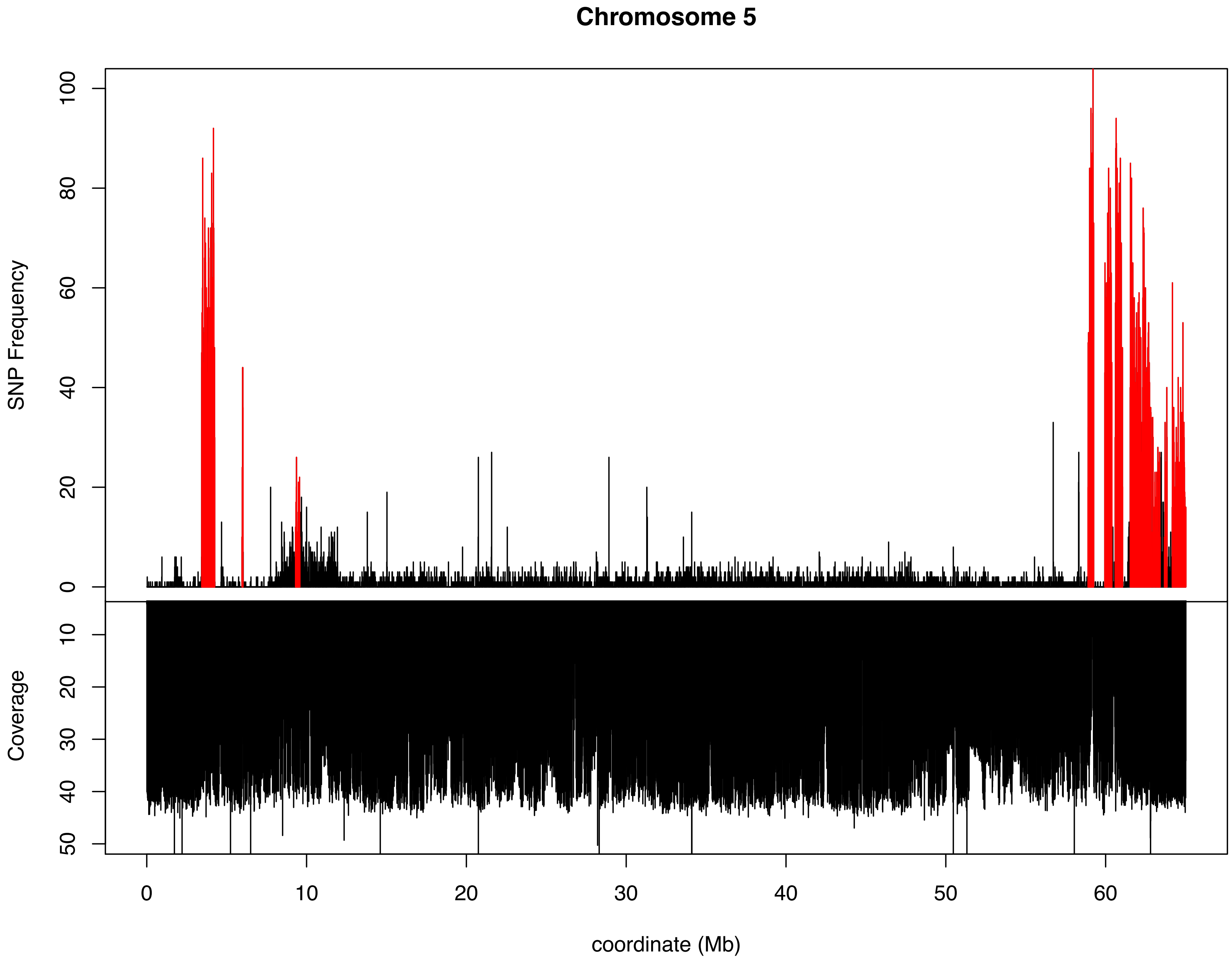
Chromosome 5
SNP Frequency
Coverage
coordinate (Mb)
0
20
40
60
80
100
10
20
30
40
50
0
10
20
30
40
50
60

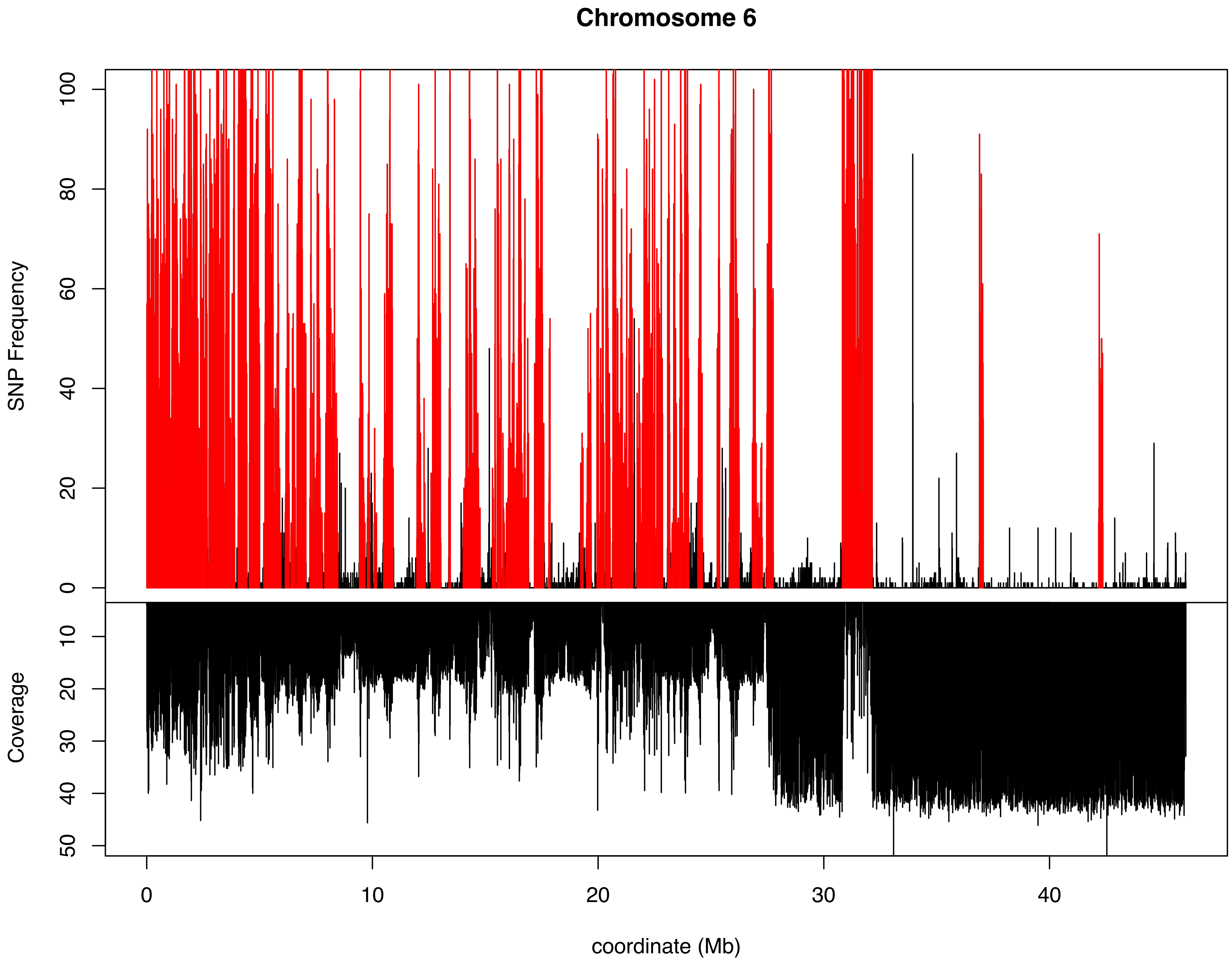
Chromosome 6
SNP Frequency
Coverage
0
20
40
60
80
100
10
20
30
40
50
0
10
20
30
40
coordinate (Mb)

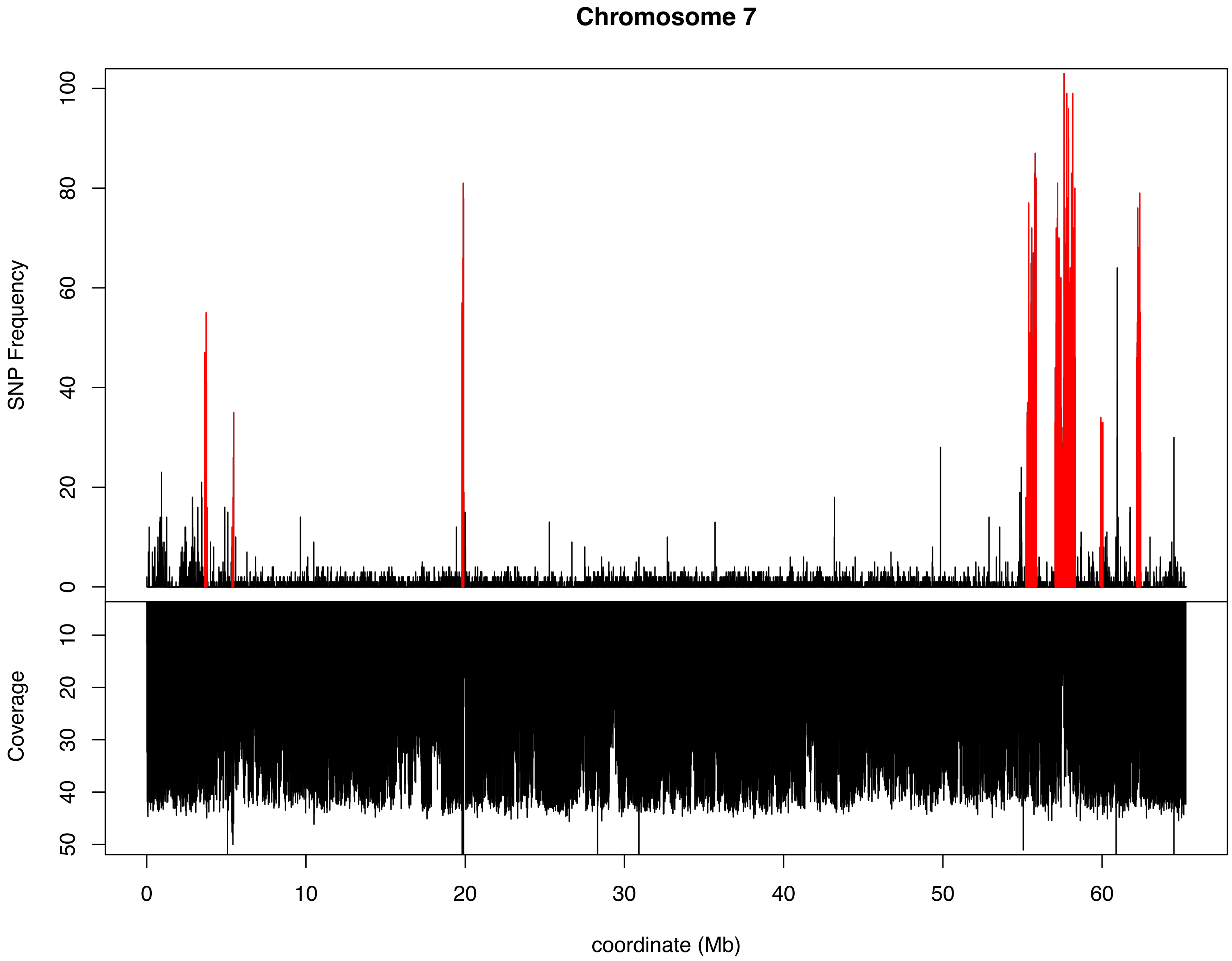
Chromosome 7
SNP Frequency
Coverage
coordinate (Mb)
0
20
40
60
80
100
10
20
30
40
50
0
10
20
30
40
50
60

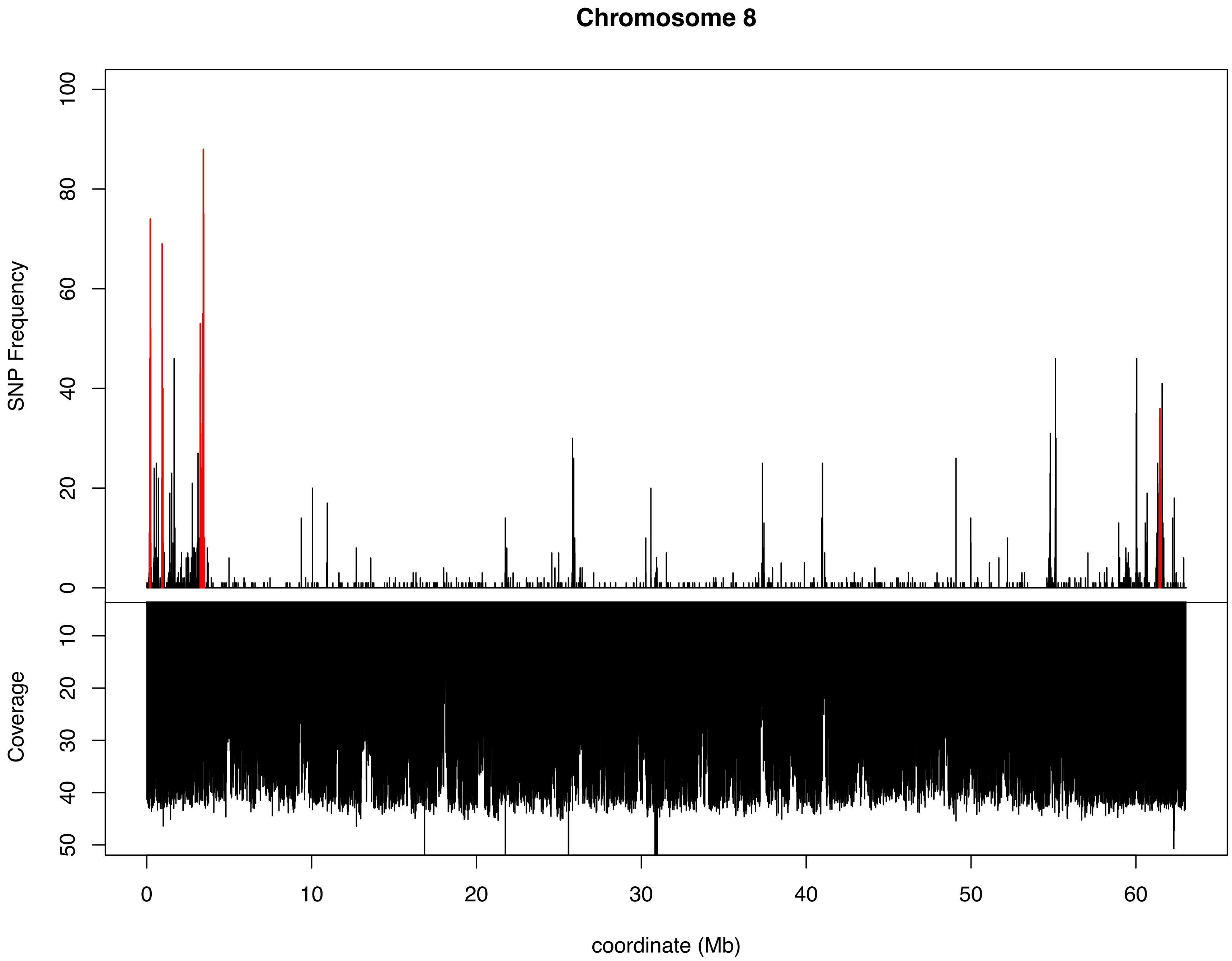
Chromosome 8
SNP Frequency
Coverage
coordinate (Mb)
0
20
40
60
80
100
10
20
30
40
50
0
10
20
30
40
50
60

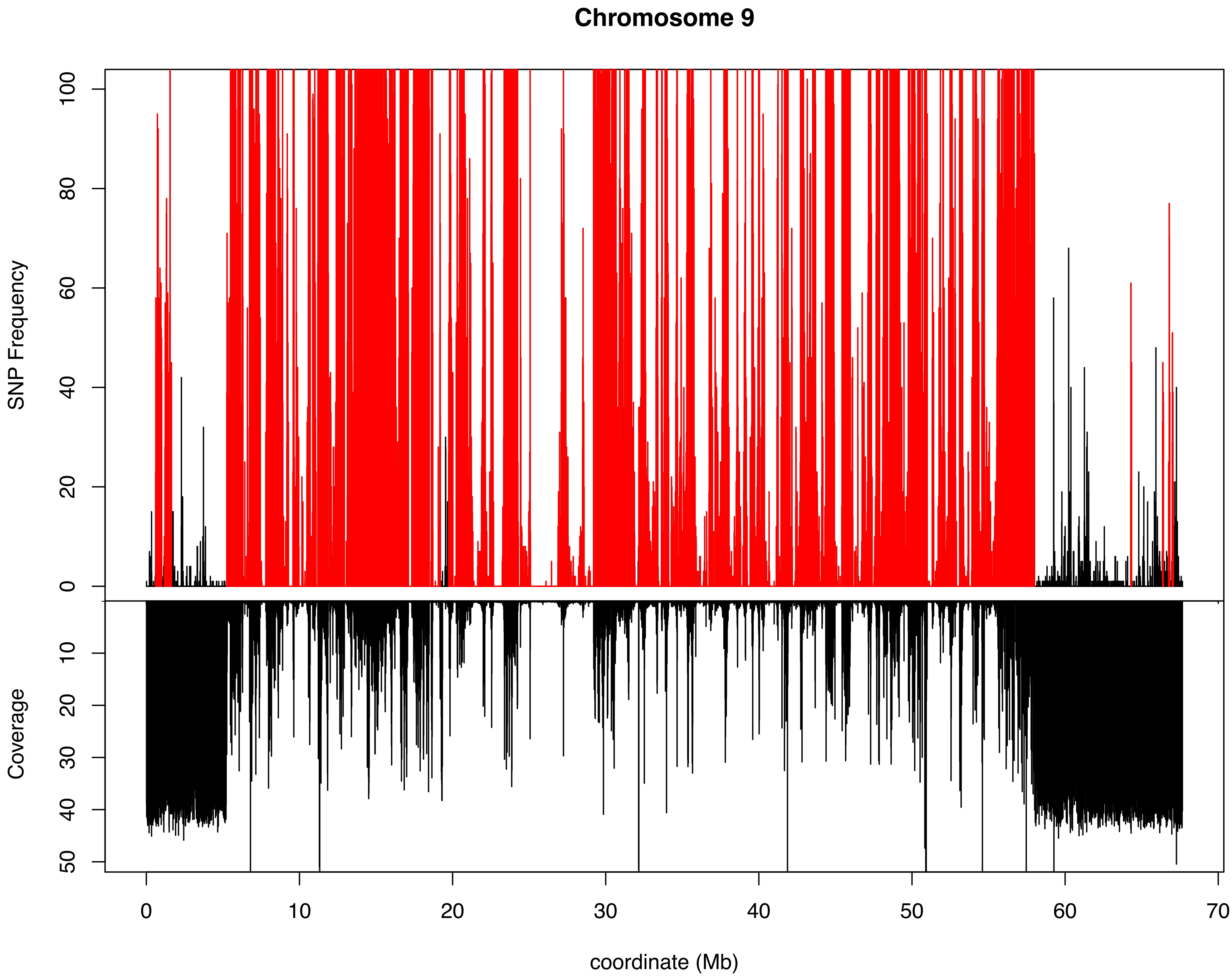

Chromosome 9
SNP Frequency
0
20
40
60
80
100
Coverage
10
20
30
40
50
0
10
20
30
40
50
60
70
coordinate (Mb)

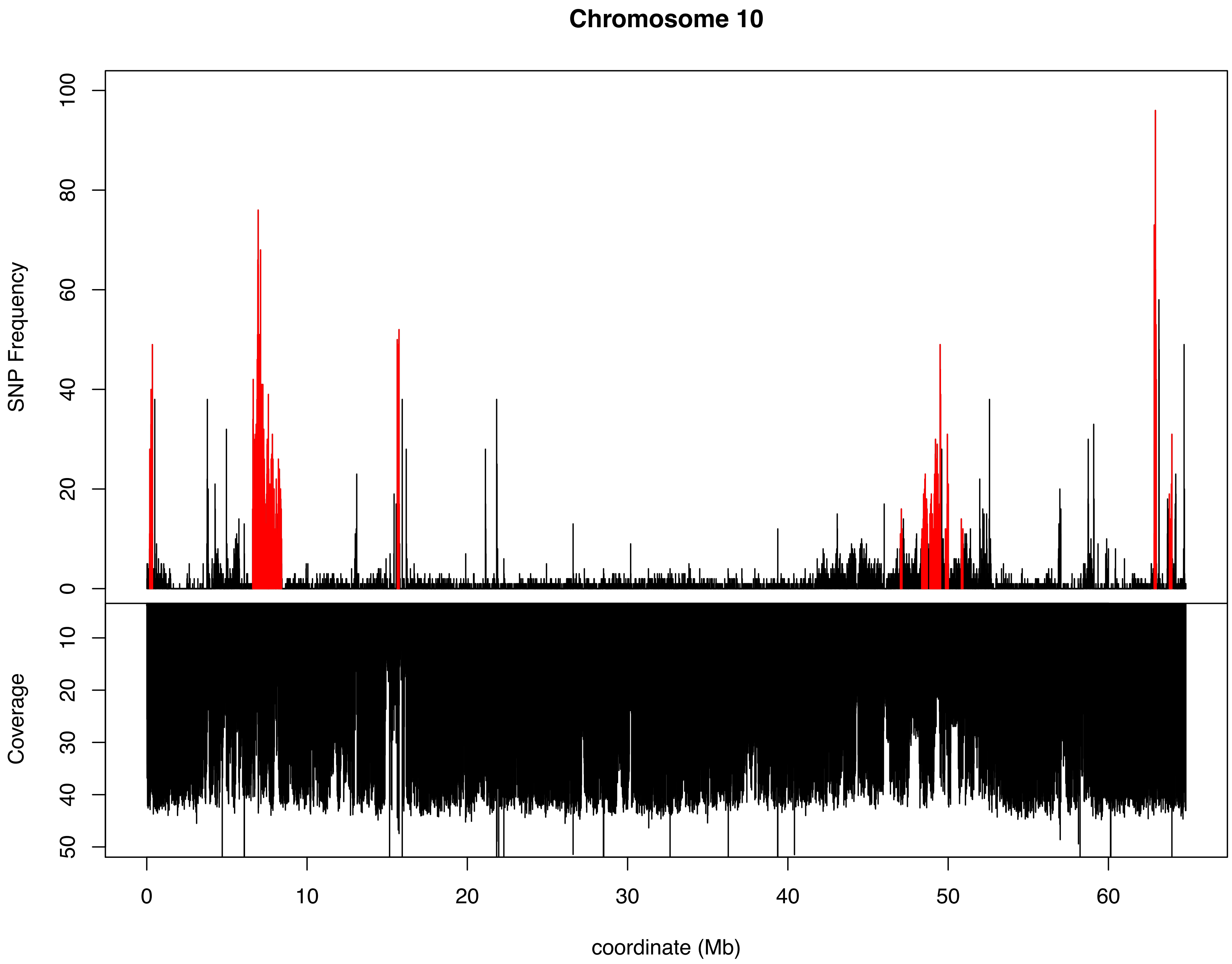

Chromosome 10
SNP Frequency
0
20
40
60
80
100
Coverage
10
20
30
40
50
0
10
20
30
40
50
60
coordinate (Mb)

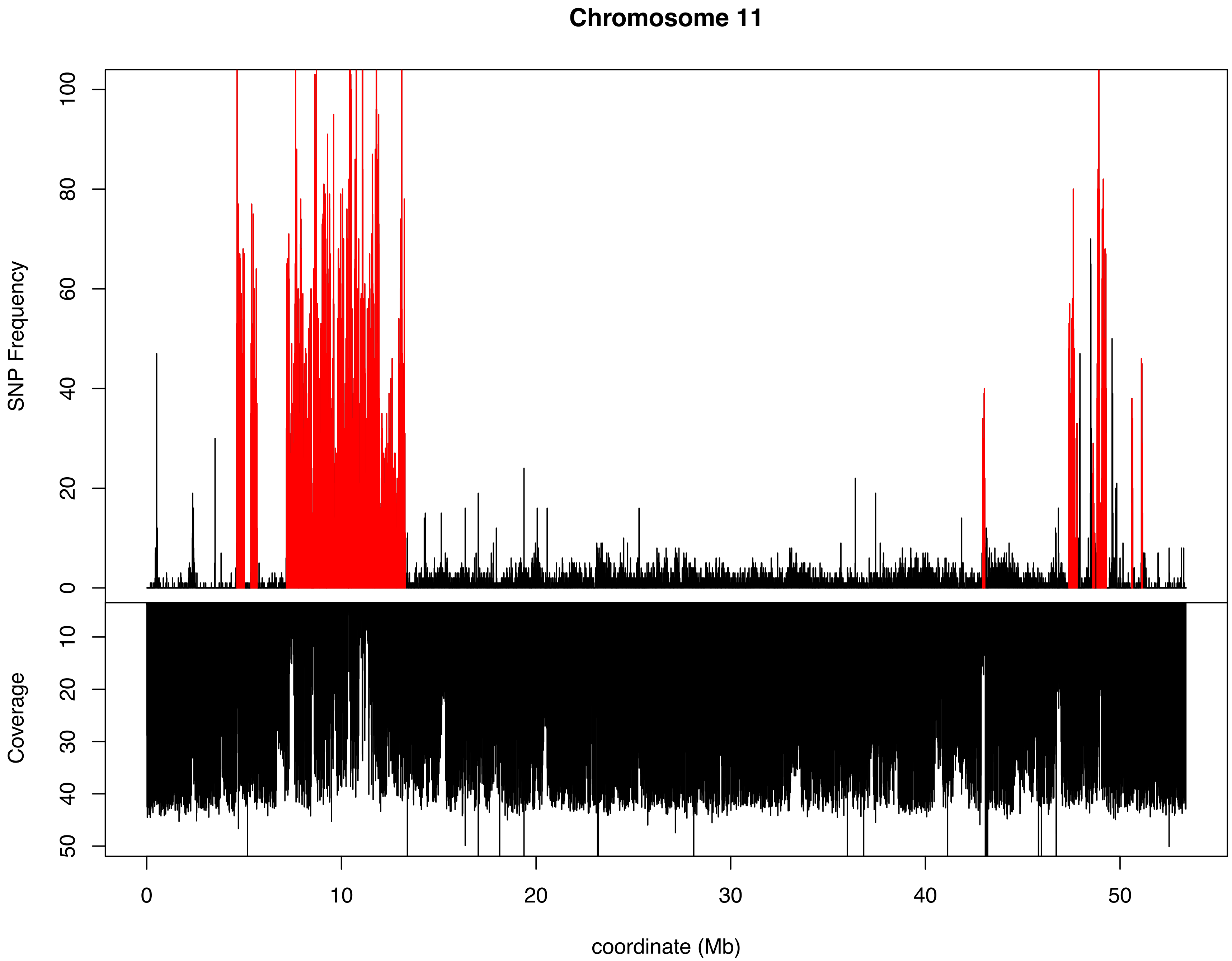

Chromosome 11
SNP Frequency
Coverage
0
20
40
60
80
100
10
20
30
40
50
0
10
20
30
40
50
coordinate (Mb)

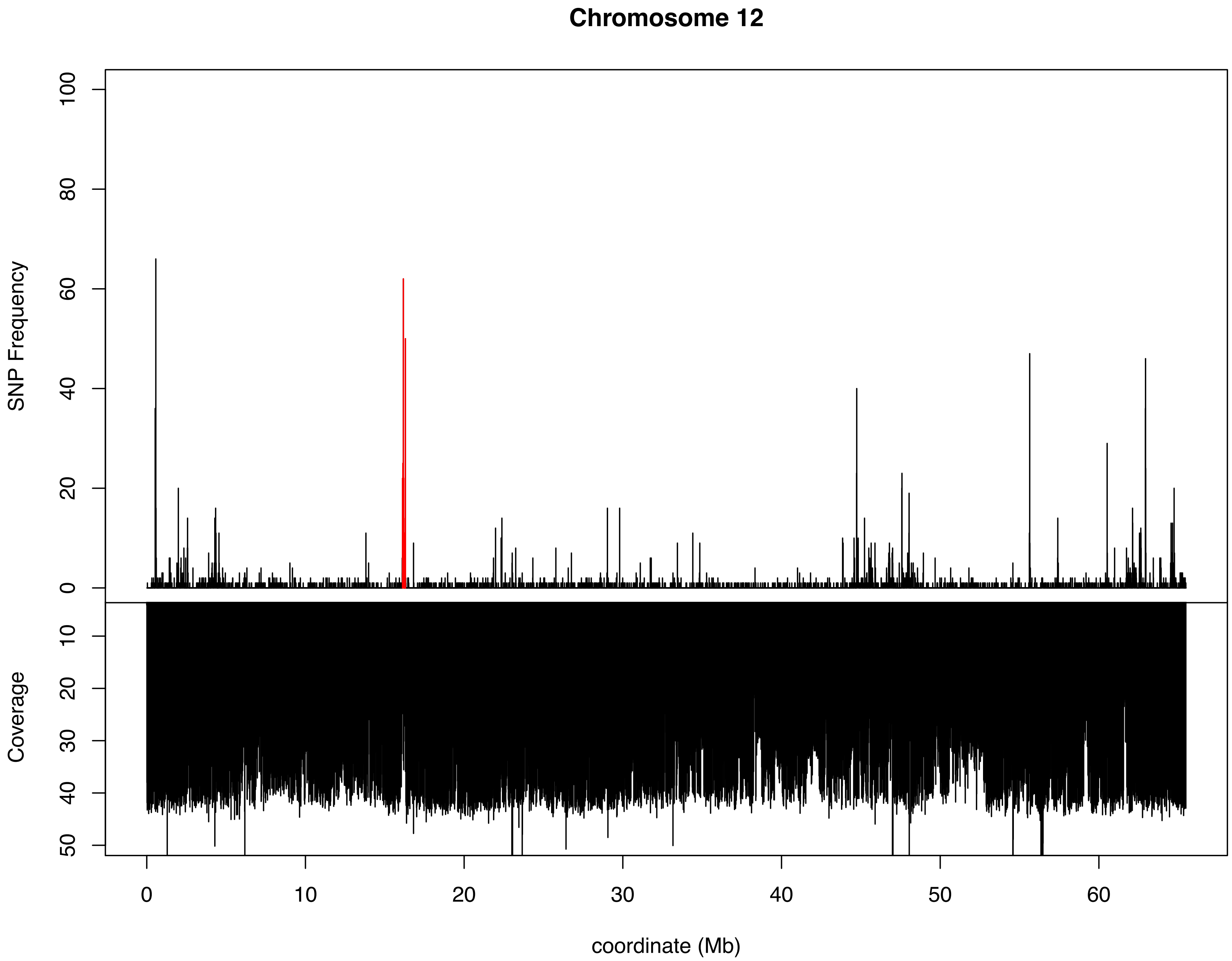
Chromosome 12
SNP Frequency
Coverage
0
20
40
60
80
100
10
20
30
40
50
0
10
20
30
40
50
60
coordinate (Mb)
