# Supplementary material for: Analysis of wild-species introgressions in tomato inbreds uncovers ancestral origins
Source: BMC Plant Biol. 2014 Oct 28;14:287. doi: 10.1186/s12870-014-0287-2 (PMC4219026; doi:10.1186/s12870-014-0287-2)
Supplement: Additional file 4: Table S2. — Summary of introgressions in BTI-87 by 10 Kb windows. [file 12870_2014_287_MOESM4_ESM.pdf]

#	chromosome	size (bp)	start	end	# of SNPs	# of tomato gene models
1	SL2.40ch01	110,000	290,001	400,000	249	13
2	SL2.40ch01	170,000	2,420,001	2,590,000	312	17
3	SL2.40ch01	150,000	4,000,001	4,150,000	376	4
4	SL2.40ch01	220,000	5,920,001	6,140,000	400	-
5	SL2.40ch01	400,000	39,550,001	39,950,000	564	5
6	SL2.40ch01	460,000	74,540,001	75,000,000	1,999	50
7	SL2.40ch01	130,000	78,400,001	78,530,000	523	18
8	SL2.40ch01	70,000	80,290,001	80,360,000	159	9
9	SL2.40ch01	50,000	81,320,001	81,370,000	157	3
10	SL2.40ch01	80,000	86,850,001	86,930,000	287	12
11	SL2.40ch01	720,000	87,660,001	88,380,000	3,179	108
12	SL2.40ch02	100,000	28,790,001	28,890,000	269	6
13	SL2.40ch02	140,000	31,850,001	31,990,000	295	19
14	SL2.40ch02	280,000	32,120,001	32,400,000	277	35
15	SL2.40ch02	140,000	33,600,001	33,740,000	179	16
16	SL2.40ch02	320,000	34,110,001	34,430,000	380	33
17	SL2.40ch02	1,000,000	34,480,001	35,480,000	3,743	141
18	SL2.40ch02	200,000	36,360,001	36,560,000	224	23
19	SL2.40ch02	520,000	36,610,001	37,130,000	566	73
20	SL2.40ch02	450,000	38,350,001	38,800,000	1,611	59
21	SL2.40ch03	70,000	1,020,001	1,090,000	267	7
22	SL2.40ch03	80,000	2,020,001	2,100,000	221	6
23	SL2.40ch03	90,000	49,700,001	49,790,000	95	4
24	SL2.40ch03	80,000	55,810,001	55,890,000	115	9
25	SL2.40ch03	200,000	56,000,001	56,200,000	449	19
26	SL2.40ch03	110,000	56,360,001	56,470,000	99	10
27	SL2.40ch03	150,000	56,520,001	56,670,000	359	15
28	SL2.40ch03	170,000	56,890,001	57,060,000	181	17
29	SL2.40ch03	1,730,000	57,110,001	58,840,000	6,629	222
30	SL2.40ch03	120,000	59,340,001	59,460,000	210	16
31	SL2.40ch03	80,000	60,760,001	60,840,000	345	14
32	SL2.40ch03	290,000	61,010,001	61,300,000	750	37
33	SL2.40ch03	50,000	61,450,001	61,500,000	137	3
34	SL2.40ch04	170,000	690,001	860,000	373	15
35	SL2.40ch04	130,000	5,250,001	5,380,000	615	13
36	SL2.40ch04	42,870,000	7,270,001	50,140,000	242,468	686
37	SL2.40ch04	90,000	54,160,001	54,250,000	437	9
38	SL2.40ch04	440,000	54,360,001	54,800,000	935	41
39	SL2.40ch04	670,000	54,850,001	55,520,000	2,086	49
40	SL2.40ch04	130,000	57,310,001	57,440,000	175	14
41	SL2.40ch04	2,130,000	57,490,001	59,620,000	8,823	217
42	SL2.40ch04	100,000	59,920,001	60,020,000	141	6
43	SL2.40ch04	430,000	61,640,001	62,070,000	2,016	54
44	SL2.40ch04	1,730,000	62,330,001	64,060,000	6,945	237
45	SL2.40ch05	820,000	3,430,001	4,250,000	3,743	75
46	SL2.40ch05	70,000	5,950,001	6,020,000	163	8
47	SL2.40ch05	280,000	9,300,001	9,580,000	312	1
48	SL2.40ch05	360,000	58,890,001	59,250,000	1,867	20
49	SL2.40ch05	450,000	59,940,001	60,390,000	1,921	38

50	SL2.40ch05	470,000	60,590,001	61,060,000	2,232	34
51	SL2.40ch05	1,350,000	61,530,001	62,880,000	4,956	163
52	SL2.40ch05	170,000	62,930,001	63,100,000	221	15
53	SL2.40ch05	260,000	63,150,001	63,410,000	323	30
54	SL2.40ch05	170,000	63,670,001	63,840,000	234	23
55	SL2.40ch05	860,000	64,160,001	65,020,000	1,680	125
56	SL2.40ch06	2,680,000	1	2,680,000	13,148	274
57	SL2.40ch06	990,000	2,730,001	3,720,000	4,704	97
58	SL2.40ch06	120,000	3,770,001	3,890,000	629	7
59	SL2.40ch06	400,000	4,040,001	4,440,000	3,457	11
60	SL2.40ch06	460,000	4,540,001	5,000,000	2,615	14
61	SL2.40ch06	770,000	5,180,001	5,950,000	2,953	13
62	SL2.40ch06	180,000	6,130,001	6,310,000	580	1
63	SL2.40ch06	220,000	6,360,001	6,580,000	415	7
64	SL2.40ch06	420,000	6,630,001	7,050,000	1,883	7
65	SL2.40ch06	550,000	7,220,001	7,770,000	1,278	6
66	SL2.40ch06	590,000	7,880,001	8,470,000	1,994	10
67	SL2.40ch06	250,000	9,420,001	9,670,000	668	3
68	SL2.40ch06	110,000	9,770,001	9,880,000	248	1
69	SL2.40ch06	110,000	10,070,001	10,180,000	119	3
70	SL2.40ch06	430,000	10,490,001	10,920,000	1,606	7
71	SL2.40ch06	370,000	11,960,001	12,330,000	923	8
72	SL2.40ch06	430,000	12,590,001	13,020,000	1,272	2
73	SL2.40ch06	70,000	13,380,001	13,450,000	323	2
74	SL2.40ch06	760,000	14,010,001	14,770,000	2,091	15
75	SL2.40ch06	140,000	15,310,001	15,450,000	251	2
76	SL2.40ch06	200,000	15,500,001	15,700,000	776	3
77	SL2.40ch06	90,000	15,750,001	15,840,000	132	-
78	SL2.40ch06	1,020,000	15,890,001	16,910,000	2,964	11
79	SL2.40ch06	420,000	17,170,001	17,590,000	1,972	5
80	SL2.40ch06	60,000	17,810,001	17,870,000	232	-
81	SL2.40ch06	110,000	19,220,001	19,330,000	135	1
82	SL2.40ch06	210,000	19,480,001	19,690,000	488	1
83	SL2.40ch06	80,000	19,940,001	20,020,000	354	4
84	SL2.40ch06	190,000	20,070,001	20,260,000	510	17
85	SL2.40ch06	220,000	20,310,001	20,530,000	787	12
86	SL2.40ch06	660,000	20,640,001	21,300,000	1,815	34
87	SL2.40ch06	180,000	21,350,001	21,530,000	459	9
88	SL2.40ch06	720,000	21,720,001	22,440,000	2,119	45
89	SL2.40ch06	360,000	22,490,001	22,850,000	1,140	19
90	SL2.40ch06	110,000	23,070,001	23,180,000	552	5
91	SL2.40ch06	230,000	23,230,001	23,460,000	618	4
92	SL2.40ch06	260,000	23,510,001	23,770,000	670	5
93	SL2.40ch06	200,000	23,820,001	24,020,000	1,436	7
94	SL2.40ch06	210,000	24,410,001	24,620,000	869	3
95	SL2.40ch06	110,000	25,270,001	25,380,000	600	4
96	SL2.40ch06	450,000	25,800,001	26,250,000	1,806	15
97	SL2.40ch06	300,000	26,830,001	27,130,000	659	5
98	SL2.40ch06	90,000	27,180,001	27,270,000	107	-
99	SL2.40ch06	290,000	27,460,001	27,750,000	1,414	18

100	SL2.40ch06	1,330,000	30,810,001	32,140,000	9,074	76
101	SL2.40ch06	170,000	36,890,001	37,060,000	844	25
102	SL2.40ch06	190,000	42,170,001	42,360,000	635	27
103	SL2.40ch07	150,000	3,630,001	3,780,000	282	14
104	SL2.40ch07	110,000	5,350,001	5,460,000	134	1
105	SL2.40ch07	120,000	19,790,001	19,910,000	556	1
106	SL2.40ch07	660,000	55,210,001	55,870,000	2,265	58
107	SL2.40ch07	1,290,000	57,040,001	58,330,000	5,307	102
108	SL2.40ch07	150,000	59,900,001	60,050,000	231	17
109	SL2.40ch07	250,000	62,170,001	62,420,000	1,078	33
110	SL2.40ch08	90,000	150,001	240,000	272	8
111	SL2.40ch08	70,000	910,001	980,000	193	8
112	SL2.40ch08	80,000	3,230,001	3,310,000	201	6
113	SL2.40ch08	130,000	3,360,001	3,490,000	328	4
114	SL2.40ch08	100,000	61,390,001	61,490,000	186	10
115	SL2.40ch09	400,000	600,001	1,000,000	1,684	57
116	SL2.40ch09	470,000	1,170,001	1,640,000	1,566	61
117	SL2.40ch09	6,890,000	5,240,001	12,130,000	27,104	191
118	SL2.40ch09	7,020,000	12,180,001	19,200,000	49,038	117
119	SL2.40ch09	12,370,000	19,730,001	32,100,000	38,854	154
120	SL2.40ch09	25,850,000	32,150,001	58,000,000	91,824	468
121	SL2.40ch09	60,000	64,280,001	64,340,000	215	5
122	SL2.40ch09	60,000	66,360,001	66,420,000	188	5
123	SL2.40ch09	90,000	66,750,001	66,840,000	242	10
124	SL2.40ch09	80,000	66,990,001	67,070,000	201	11
125	SL2.40ch10	190,000	170,001	360,000	335	26
126	SL2.40ch10	1,360,000	6,600,001	7,960,000	3,234	33
127	SL2.40ch10	110,000	8,010,001	8,120,000	129	2
128	SL2.40ch10	240,000	8,170,001	8,410,000	359	2
129	SL2.40ch10	130,000	15,620,001	15,750,000	250	-
130	SL2.40ch10	110,000	47,010,001	47,120,000	91	3
131	SL2.40ch10	360,000	48,340,001	48,700,000	446	16
132	SL2.40ch10	680,000	48,850,001	49,530,000	964	11
133	SL2.40ch10	190,000	49,820,001	50,010,000	189	6
134	SL2.40ch10	110,000	50,810,001	50,920,000	94	3
135	SL2.40ch10	140,000	62,840,001	62,980,000	661	20
136	SL2.40ch10	170,000	63,790,001	63,960,000	163	27
137	SL2.40ch11	390,000	4,620,001	5,010,000	1,749	51
138	SL2.40ch11	330,000	5,340,001	5,670,000	1,374	36
139	SL2.40ch11	6,120,000	7,170,001	13,290,000	22,504	220
140	SL2.40ch11	120,000	42,930,001	43,050,000	255	4
141	SL2.40ch11	430,000	47,360,001	47,790,000	1,316	35
142	SL2.40ch11	110,000	48,590,001	48,700,000	153	6
143	SL2.40ch11	480,000	48,810,001	49,290,000	2,044	40
144	SL2.40ch11	60,000	50,590,001	50,650,000	164	4
145	SL2.40ch11	60,000	51,090,001	51,150,000	171	3
146	SL2.40ch12	190,000	16,110,001	16,300,000	264	3
	**Total:**	**150,160,000**			**641,454**	**5,633**
