# Supplementary material for: Analysis of wild-species introgressions in tomato inbreds uncovers ancestral origins
Source: BMC Plant Biol. 2014 Oct 28;14:287. doi: 10.1186/s12870-014-0287-2 (PMC4219026; doi:10.1186/s12870-014-0287-2)
Supplement: Additional file 5: Table S3. — Gh13 introgressions summary and SolCAP introgression regions. [file 12870_2014_287_MOESM5_ESM.pdf]

	SNP density regions GH13 x Heinz (WGS)						Gh13 x HUJ (SolCAP)			
chromosome	size (bp)	Start	End	# of tomato gene models WGS region	Number of SNPs in Gh13 introgression	Predicted introgression (origin)	Start	End	# nt in introgression SolCAP	number of polymorphic SolCAP markers (monomorphic)
SL2.40ch01	60,000	290,001	350,000	9	147	Heinz (unknown)				
SL2.40ch01	60,000	650,001	710,000	3	66	Gh13 (Unknown)				
SL2.40ch01	170,000	2,420,001	2,590,000	17	361	Gh13 and Heinz (unknown)	2,255,882	2,463,253	207,371	14 (1)
SL2.40ch01	150,000	4,000,001	4,150,000	4	406	Heinz (unknown)				
SL2.40ch01	60,000	4,940,001	5,000,000	5	272	Gh13 (Unknown)				
SL2.40ch01	220,000	5,920,001	6,140,000	0	889	Heinz (unknown)				
SL2.40ch01	340,000	10,680,001	11,020,000	4	890	Heinz (unknown)				
SL2.40ch01	100,000	23,870,001	23,970,000	42	94	Gh13 and Heinz (unknown)				
SL2.40ch01	120,000	37,240,001	37,360,000	2	180	Gh13 and Heinz (unknown)				
SL2.40ch01	400,000	39,550,001	39,950,000	5	619	Heinz (unknown)				
SL2.40ch01							70,262,679	70,579,788	317,109	5 (0)
SL2.40ch01							72,796,829	73,233,146	436,317	8 (1)
SL2.40ch01	70,000	80,290,001	80,360,000	9	159	Heinz (unknown)				
SL2.40ch01	50,000	81,320,001	81,370,000	3	155	Heinz (unknown)				
SL2.40ch01	80,000	86,850,001	86,930,000	12	270	Heinz (unknown)				
SL2.40ch01	80,000	90,220,001	90,300,000	5	329	Heinz (unknown)				
SL2.40ch02	70,000	4,030,001	4,100,000	0	209	Heinz (unknown)				
SL2.40ch02	110,000	4,980,001	5,090,000	18	128	Gh13 (Unknown)				
SL2.40ch02	160,000	20,590,001	20,750,000	2	216	Heinz (unknown)				
SL2.40ch02	80,000	27,500,001	27,580,000	1	140	Heinz (unknown)				
SL2.40ch02	290,000	31,510,001	31,800,000	18	708	Heinz (unknown)				
SL2.40ch02	140,000	31,850,001	31,990,000	19	297	Heinz (unknown)	31,567,643	31,920,553	352,910	11 (4)
SL2.40ch02	200,000	32,200,001	32,400,000	25	227	Heinz (unknown)				
SL2.40ch02	50,000	34,670,001	34,720,000	10	92	Heinz (unknown)				
SL2.40ch02							34,096,831	34,388,704	291,873	9 (1)
SL2.40ch02	50,000	37,370,001	37,420,000	2	110	Gh13 (Unknown)				
SL2.40ch02							39,890,909	39,907,966	17,057	13 (0)
SL2.40ch03							71,076	828,684	757,608	8 (6)
SL2.40ch03							7,085,127	7,095,859	10,732	3 (0)
SL2.40ch03	70,000	1,020,001	1,090,000	7	254	Gh13 and Heinz (unknown)				

SL2.40ch03	60,000	1,250,001	1,310,000	8	112	Gh13 and Heinz (unknown)				
SL2.40ch03	80,000	2,020,001	2,100,000	6	212	Heinz (unknown)				
SL2.40ch03	310,000	10,730,001	11,040,000	11	413	Heinz (unknown)				
SL2.40ch03	140,000	17,260,001	17,400,000	1	334	Heinz (unknown)				
SL2.40ch03	90,000	45,550,001	45,640,000	8	222	Gh13 (Unknown)	45,591,053	45,630,131	39,078	11 (0)
SL2.40ch03	50,000	48,100,001	48,150,000	2	180	Heinz (unknown)				
SL2.40ch03	60,000	49,700,001	49,760,000	1	77	Gh13 (Unknown)				
SL2.40ch03	90,000	56,100,001	56,190,000	8	262	Heinz (unknown)				
SL2.40ch03							57,030,423	57,366,629	336,206	11 (5)
SL2.40ch03							57,671,208	58,803,206	1,131,998	12 (6)
SL2.40ch03	120,000	59,340,001	59,460,000	16	194	Gh13 (Unknown)				
SL2.40ch03							60,773,120	60,818,312	45,192	29 (1)
SL2.40ch04	90,000	690,001	780,000	8	187	Heinz (unknown)	744,896	993,580	248,684	14 (9)
SL2.40ch04	250,000	4,470,001	4,720,000	29	279	Heinz (unknown)	4,635,914	4,647,789	11,875	3 (1)
SL2.40ch04	690,000	28,670,001	29,360,000	2	1,207	Heinz (unknown)				
SL2.40ch04	2,170,000	53,350,001	55,520,000	158	11,030	Heinz (200 Kb unknown)				
SL2.40ch04	130,000	57,310,001	57,440,000	14	168	Heinz (unknown)				
SL2.40ch04	2,150,000	57,490,001	59,640,000	220	8,555	pimpinellifolium)				
SL2.40ch04	100,000	59,920,001	60,020,000	6	149	Heinz (unknown)				
SL2.40ch04	140,000	60,520,001	60,660,000	21	179	Gh13 (Unknown)				
SL2.40ch05							734,561	736,089	1,528	5 (0)
SL2.40ch05							3,437,023	4,244,925	807,902	105 (6)
SL2.40ch05	80,000	5,950,001	6,030,000	9	187	Heinz (unknown)				
SL2.40ch05	780,000	8,930,001	9,710,000	26	1,061	Gh13 (Unknown)				
SL2.40ch05	180,000	59,250,001	59,430,000	10	802	Heinz (unknown)	7,473,754	59,429,177	51,955,423	126 (34)
SL2.40ch05	450,000	59,940,001	60,390,000	38	2,072	Heinz (unknown)				
SL2.40ch05							60,639,488	61,040,201	400,713	38 (1)
SL2.40ch05	110,000	62,340,001	62,450,000	13	118	Heinz (unknown)	61,927,092	62,644,894	717,802	67 (3)
SL2.40ch05	50,000	62,950,001	63,000,000	3	140	Gh13 (Unknown)				
SL2.40ch06	160,000	1,600,001	1,760,000	25	337					
SL2.40ch06						Gh13 and Heinz (unknown)	2,083,443	2,386,312	302,869	8 (5)
SL2.40ch06	80,000	14,660,001	14,740,000	6	220	Heinz (unknown)				
SL2.40ch06	150,000	15,230,001	15,380,000	2	241	Heinz (unknown)				
SL2.40ch06	3,620,000	30,600,001	34,220,000	284	24,831	Gh13 (S. chilense)	30,623,784	33,972,993	3,349,209	29 (10)
SL2.40ch07							1,815,826	2,131,288	315,462	7 (4)
SL2.40ch07	150,000	3,630,001	3,780,000	14	310	Gh13 and Heinz (unknown)				
SL2.40ch07	170,000	5,290,001	5,460,000	3	318	Heinz (unknown)				
SL2.40ch07	100,000	6,750,001	6,850,000	4	157	Heinz (unknown)				

SL2.40ch07	120,000	19,790,001	19,910,000	1	521	Heinz (unknown)				
SL2.40ch07	60,000	54,900,001	54,960,000	4	220	Heinz (unknown)				
SL2.40ch07	1,290,000	57,040,001	58,330,000	102	5,899	Gh13 (Unknown)	57,047,892	59,386,207	2,338,315	44 (15)
SL2.40ch07	190,000	59,860,001	60,050,000	21	240	Gh13 and Heinz (unknown)				
SL2.40ch07	100,000	60,540,001	60,640,000	13	95	Gh13 (Unknown)				
SL2.40ch07	50,000	60,930,001	60,980,000	7	248	Gh13 and Heinz (unknown)				
SL2.40ch07	130,000	62,700,001	62,830,000	15	548	Gh13 (S. pimpinellifolium)	62,722,518	62,821,354	98,836	7 (2)
SL2.40ch08							35,179	236,771	201,592	4 (2)
SL2.40ch08	60,000	180,001	240,000	5	232	Heinz (unknown)				
SL2.40ch08	70,000	910,001	980,000	8	193	Heinz (unknown)				
SL2.40ch08	50,000	1,280,001	1,330,000	2	176	Gh13 (Unknown)				
SL2.40ch08	110,000	2,840,001	2,950,000	11	110	Gh13 and Heinz (unknown)				
SL2.40ch08	80,000	3,230,001	3,310,000	6	172	Heinz (unknown)				
SL2.40ch08	130,000	3,360,001	3,490,000	4	317	Heinz (unknown)	2,580,771	3,422,383	841,612	11 (11)
SL2.40ch08	150,000	25,820,001	25,970,000	1	168	Heinz (unknown)				
SL2.40ch08	60,000	40,940,001	41,000,000	1	117	Heinz (unknown)				
SL2.40ch08	70,000	55,080,001	55,150,000	12	122	Gh13 (Unknown)	55,108,214	55,124,760	16,546	7 (0)
SL2.40ch08	90,000	55,560,001	55,650,000	7	566	Gh13 and Heinz (unknown)				
SL2.40ch08	70,000	59,990,001	60,060,000	8	132	Heinz (unknown)	60,016,886	60,467,067	450,181	7 (1)
SL2.40ch08	90,000	61,400,001	61,490,000	9	160	Heinz (unknown)	61,303,467	61,402,166	98,699	6 (1)
SL2.40ch09	330,000	330,001	660,000	46	696	Gh13 and Heinz (unknown)	41,189	651,775	610,586	24 (2)
SL2.40ch09	110,000	1,530,001	1,640,000	12	347	Heinz (unknown)				
SL2.40ch09	120,000	2,290,001	2,410,000	17	121	Gh13 and Heinz (unknown)				
SL2.40ch09							60,765,597	61,511,672	746,075	3 (5)
SL2.40ch09	350,000	63,180,001	63,530,000	37	538	Gh13 (Unknown)				
SL2.40ch09	130,000	65,130,001	65,260,000	12	239	Gh13 (Unknown)	65,153,501	65,211,528	58,027	4 (0)
SL2.40ch09	60,000	66,360,001	66,420,000	5	194	Gh13 (Unknown)				
SL2.40ch09	90,000	66,750,001	66,840,000	10	233	Gh13 (Unknown)				
SL2.40ch09	80,000	66,990,001	67,070,000	11	225	Gh13 (Unknown)				
SL2.40ch10							889,234	1,163,895	274,661	4 (2)
SL2.40ch10							3,017,289	3,092,679	75,390	3 (0)
SL2.40ch10	140,000	5,530,001	5,670,000	0	128	Heinz (unknown)				
SL2.40ch10	1,790,000	6,600,001	8,390,000	39	4,652	Heinz (unknown)				
SL2.40ch10	170,000	15,620,001	15,790,000	0	268	Heinz (unknown)				
SL2.40ch10	90,000	21,800,001	21,890,000	3	247	Heinz (unknown)				
SL2.40ch10	220,000	47,010,001	47,230,000	8	204	Heinz (unknown)				
SL2.40ch10	330,000	47,700,001	48,030,000	4	358	Heinz (unknown)				
SL2.40ch10	410,000	48,330,001	48,740,000	18	591	Heinz (unknown)				

SL2.40ch10	950,000	48,790,001	49,740,000	21	1,695	Heinz (unknown)				
SL2.40ch10	220,000	49,790,001	50,010,000	6	311	Heinz (unknown)				
SL2.40ch10	170,000	50,180,001	50,350,000	3	145	Heinz (unknown)				
SL2.40ch10	160,000	50,400,001	50,560,000	4	129	Heinz (unknown)				
SL2.40ch10	370,000	50,770,001	51,140,000	17	330	Heinz (unknown)				
SL2.40ch10	160,000	51,250,001	51,410,000	5	143	Heinz (unknown)				
SL2.40ch10	160,000	56,870,001	57,030,000	5	531	Gh13 and Heinz (unknown)				
SL2.40ch10							59,641,345	59,988,884	347,539	4 (4)
SL2.40ch10	170,000	63,790,001	63,960,000	27	238	Gh13 and Heinz (unknown)				
SL2.40ch10							64,133,464	64,199,335	65,871	3 (1)
SL2.40ch11							436,089	791,672	355,583	8 (6)
SL2.40ch11	70,000	2,330,001	2,400,000	0	98	400 Kb)				
SL2.40ch11	440,000	4,570,001	5,010,000	55	1,726	Gh13 (S. pimpinellifolium ?)	4,775,241	5,471,885	696,644	39 (8)
SL2.40ch11	180,000	7,590,001	7,770,000	5	982	Gh13 (Unknown)	7,763,703	7,863,337	99,634	3 (0)
SL2.40ch11	100,000	8,010,001	8,110,000	6	156	Gh13 (Unknown)				
SL2.40ch11	170,000	8,160,001	8,330,000	10	412	Gh13 (Unknown)				
SL2.40ch11	70,000	8,450,001	8,520,000	0	107	Gh13 and Heinz (unknown)				
SL2.40ch11	100,000	9,930,001	10,030,000	6	188	Gh13 (Unknown)				
SL2.40ch11	11,760,000	23,180,001	34,940,000	162	49,402	Gh13 (S. pimpinellifolium)	8,163,278	25,080,735	16,917,457	92 (12)
SL2.40ch11							34,946,763	40,370,165	5,423,402	49 (3)
SL2.40ch11	230,000	42,900,001	43,130,000	23	423	Gh13 and Heinz (unknown)				
SL2.40ch11	4,490,000	43,180,001	47,670,000	172	21,080	Gh13 (S. pimpinellifolium)				
SL2.40ch11	60,000	48,470,001	48,530,000	4	192	Gh13 (Unknown)				
SL2.40ch11	70,000	48,580,001	48,650,000	3	154	Gh13 (Unknown)				
SL2.40ch11	130,000	48,810,001	48,940,000	11	213	Gh13 (Unknown)	48,827,340	49,276,373	449,033	44 (2)
SL2.40ch11	60,000	50,590,001	50,650,000	4	144	Gh13 (Unknown)	50,619,142	50,643,804	24,662	3 (0)
SL2.40ch11	60,000	51,090,001	51,150,000	3	153	Gh13 (Unknown)				
SL2.40ch11	290,000	51,930,001	52,220,000	39	1077	Gh13 (Unknown)	51,935,919	52,154,898	218,979	36 (7)
SL2.40ch12							3,977,225	4,038,812	61,587	5 (0)
SL2.40ch12	140,000	4,280,001	4,420,000	13	287	Gh13 and Heinz (unknown)				
SL2.40ch12	100,000	4,600,001	4,700,000	7	508	Gh13 (S. pimpinellifolium)	4,638,580	5,069,448	430,868	3 (2)
SL2.40ch12	100,000	4,950,001	5,050,000	6	328	Gh13 (S. pimpinellifolium)				
SL2.40ch12	190,000	5,100,001	5,290,000	9	553	Gh13 (S. pimpinellifolium)	5,158,348	6,682,212	1,523,864	5 (12)
SL2.40ch12	180,000	8,220,001	8,400,000	0	153	Gh13 and Heinz (unknown)				
SL2.40ch12	170,000	13,450,001	13,620,000	0	135	Gh13 and Heinz (unknown)				
SL2.40ch12	220,000	16,080,001	16,300,000	3	617	Gh13 and Heinz (unknown)				
SL2.40ch12	180,000	18,480,001	18,660,000	1	159	Gh13 and Heinz (unknown)				
SL2.40ch12	330,000	22,540,001	22,870,000	4	1,367	Gh13 and Heinz (unknown)				

SL2.40ch12	200,000	26,790,001	26,990,000	0	191	Gh13 and Heinz (unknown)				
SL2.40ch12	230,000	27,710,001	27,940,000	0	222	Gh13 and Heinz (unknown)				
SL2.40ch12	130,000	33,890,001	34,020,000	5	133	Gh13 and Heinz (unknown)				
SL2.40ch12	120,000	34,900,001	35,020,000	2	136	Gh13 and Heinz (unknown)				
SL2.40ch12	90,000	36,610,001	36,700,000	1	85	Gh13 and Heinz (unknown)				
SL2.40ch12	120,000	37,200,001	37,320,000	0	122	Gh13 and Heinz (unknown)				
SL2.40ch12	100,000	37,780,001	37,880,000	0	96	Gh13 and Heinz (unknown)				
SL2.40ch12	290,000	38,160,001	38,450,000	5	307	Gh13 and Heinz (unknown)				
SL2.40ch12	170,000	41,690,001	41,860,000	0	145	Gh13 and Heinz (unknown)				
SL2.40ch12	110,000	42,190,001	42,300,000	1	92	Gh13 and Heinz (unknown)				
SL2.40ch12	380,000	42,730,001	43,110,000	17	358	Gh13 and Heinz (unknown)				
SL2.40ch12	60,000	43,720,001	43,780,000	3	71	Heinz (unknown)				
SL2.40ch12	330,000	43,830,001	44,160,000	8	1,711	Gh13 and Heinz (unknown)				
SL2.40ch12	70,000	46,950,001	47,020,000	3	218	Gh13 and Heinz (unknown)	45,650,254	47,221,911	1,571,657	6 (8)
SL2.40ch12	80,000	50,560,001	50,640,000	1	76	Gh13 and Heinz (unknown)				
SL2.40ch12	250,000	50,690,001	50,940,000	0	205	Gh13 and Heinz (unknown)				
SL2.40ch12	50,000	52,270,001	52,320,000	0	502	Gh13 and Heinz (unknown)				
SL2.40ch12	170,000	54,070,001	54,240,000	2	829	Gh13 and Heinz (unknown)				
SL2.40ch12	70,000	55,610,001	55,680,000	2	172	Gh13 and Heinz (unknown)				
SL2.40ch12	80,000	56,390,001	56,470,000	0	77	Gh13 and Heinz (unknown)				
SL2.40ch12	190,000	57,840,001	58,030,000	0	160	Gh13 and Heinz (unknown)				
SL2.40ch12	160,000	59,150,001	59,310,000	0	134	Gh13 and Heinz (unknown)				
SL2.40ch12	350,000	61,390,001	61,740,000	5	484	Gh13 and Heinz (unknown)				
SL2.40ch12							62,436,776	63,147,538	710,762	8 (15)
**coverage:**	**49,420,000**		**genes:**	**2,326**	**171,711**				**96,762,980**	**968**
